# 3D Printing to Increase the Flexibility of the Chemical Synthesis of Biologically Active Molecules: Design of On-Demand Gas Generation Reactors

**DOI:** 10.3390/ijms22189919

**Published:** 2021-09-14

**Authors:** Kirill S. Erokhin, Evgeniy G. Gordeev, Dmitriy E. Samoylenko, Konstantin S. Rodygin, Valentine P. Ananikov

**Affiliations:** 1 N. D. Zelinsky Institute of Organic Chemistry Russian Academy of Sciences, Leninsky Prospect 47, 119991 Moscow, Russia; erokhin@ioc.ac.ru (K.S.E.); gordeev_e@ioc.ac.ru (E.G.G.); k.rodygin@spbu.ru (K.S.R.); 2 Institute of Chemistry, Saint Petersburg State University, Universitetsky Prospect 26, 198504 Peterhof, Russia; d.samoylenko@spbu.ru

**Keywords:** 3D printing, additive manufacturing, acetylene, carbon dioxide, hydrogen, organic synthesis

## Abstract

The development of new drugs is accelerated by rapid access to functionalized and D-labeled molecules with improved activity and pharmacokinetic profiles. Diverse synthetic procedures often involve the usage of gaseous reagents, which can be a difficult task due to the requirement of a dedicated laboratory setup. Here, we developed a special reactor for the on-demand production of gases actively utilized in organic synthesis (C_2_H_2_, H_2_, C_2_D_2_, D_2_, and CO_2_) that completely eliminates the need for high-pressure equipment and allows for integrating gas generation into advanced laboratory practice. The reactor was developed by computer-aided design and manufactured using a conventional 3D printer with polypropylene and nylon filled with carbon fibers as materials. The implementation of the reactor was demonstrated in representative reactions with acetylene, such as atom-economic nucleophilic addition (conversions of 19–99%) and nickel-catalyzed S-functionalization (yields 74–99%). One of the most important advantages of the reactor is the ability to generate deuterated acetylene (C_2_D_2_) and deuterium gas (D_2_), which was used for highly significant, atom-economic and cost-efficient deuterium labeling of S,O-vinyl derivatives (yield 68–94%). Successful examples of their use in organic synthesis are provided to synthesize building blocks of heteroatom-functionalized and D-labeled biologically active organic molecules.

## 1. Introduction

The development of new drugs urgently requires accelerated chemical synthesis procedures to access key molecular building blocks and synthesize libraries of new molecules. Modular synthetic procedures based on simple starting materials are demanded the most to achieve cost-efficient and rapid preparation of new organic molecules. Heteroatom-functionalized molecules are the key substances for modern pharma, and a new wave is stimulated by exploring deuterium-labeled (D-labeled) molecules, which show an improved pharmacokinetic profile. The use of acetylenic starting materials is one of the most efficient ways to achieve cost-efficient functionalization in eco-friendly organic synthesis with reduced generation of wastes.

Acetylene is one of the most important building blocks in organic synthesis due to its availability and the many transformations developed, such as vinylation [1], carbonylation [2,3], Favorsky reactions [4], and coupling reactions [5], among many others [6,7,8]. Advances in acetylene chemistry have recently been highlighted and have shown an emerging growing trend [6,9]. The transformations of acetylene have been implemented in industrial processes [10] and used in the development of a new generation of smart multifunctional materials [11,12].

**Figure 1:**
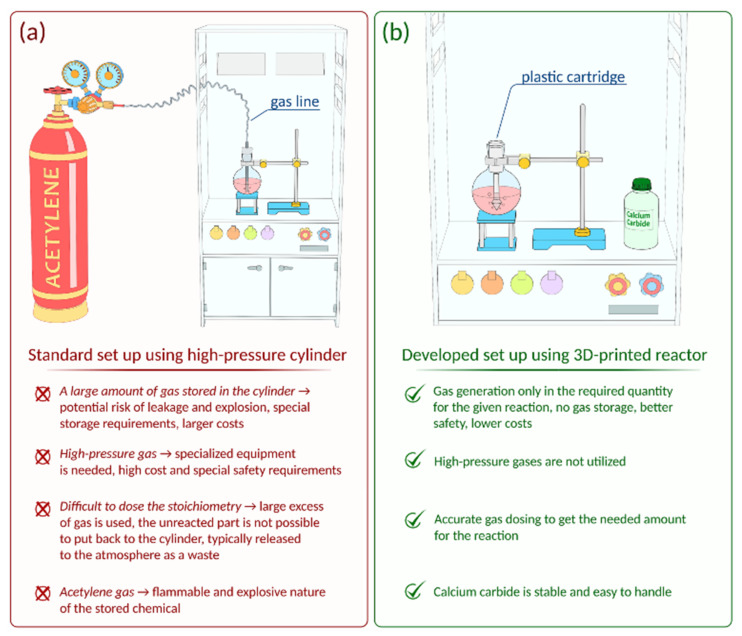
(**a**) Usage of a high-pressure acetylene cylinder (a standard approach); (**b**) application of a 3D printed on-demand acetylene generator (i.e., acetylene cartridge) without gas storage (cylinder-free approach, used in this work).

However, the flammability and explosiveness of gaseous acetylene limits the scope of possible transformations compared to other alkynes. Special safety requirements should be followed, and dedicated specialized laboratory equipment (gas lines, gas cylinders, valves, etc.) is required for successful reactions with acetylene gas (Figure 1a). The use of such equipment involves a risk of leakage. Acetylene can often be stored in acetone solution in high-pressure gas cylinders; working with it also requires compliance with safety precautions.

Hydrogen and CO_2_ are essential reagents that are widely used for hydrogenation [13,14,15] and carboxylation [16,17,18,19] reactions, both in industry and laboratory practice. Each of the gases requires dedicated equipment to involve them in a diverse range of reactions.

The approach developed here is based on two key components: the use of on-demand production of gas from a suitable precursor and the design of the reactor using 3D printing. The precursor for acetylene is calcium carbide, powerful potential of which has recently been studied through a series of chemical transformations, where acetylene is formed by the addition of water [20,21,22,23,24,25,26,27,28,29,30,31,32,33,34,35,36,37,38]. However, the presence of calcium carbide, water, and calcium hydroxide (a side product) is not compatible with a large number of water- or base-sensitive chemical reactions. The same issue concerns the generation of other gases (CO_2_ and H_2_). Therefore, the formation of gaseous reagents from precursors must be spatially separated to avoid interfering with the chemical process. We found that the design of the reactor for acetylene generation using 3D printing technology provides a complete solution to this complex problem.

Currently, additive manufacturing (AM), or 3D printing, is widely applied in many areas of science and technology, such as medicine [39,40,41,42,43,44], engineering [45,46,47,48], and material sciences [49,50,51,52,53]. In chemistry, 3D printing improves process efficiency by manufacturing fluidics [54,55,56], customized reactors for organic synthesis [57,58,59,60,61], and specific catalytic devices [62,63,64]. Key benefits of additive manufacturing include rapid prototyping and the creation of products with a complex architecture, the manufacture of which would be laborious or even impossible by classical methods [65]. AM involves several approaches potentially applicable in reactor design. Material extrusion (particularly, fused filament fabrication or FFF) stands out among them due to the low cost of personal 3D printers. Along with the wide possibilities of 3D printing for creating products of complex shapes, the FFF method is distinguished by the possibility of using it directly in the chemical laboratory, which is important since it significantly speeds up the optimization of product design. In addition, the FFF method allows for the use of a variety of materials, in particular polypropylene (PP) [66]. PP is available for 3D printing, while the production of complex PP parts by classical methods (turning and milling) requires significant costs and bulky equipment. For chemistry purposes, 3D printing allows for the design of reactors and devices to improve reaction efficiency [67,68,69,70,71,72,73].

Undoubtedly, customized gas generating equipment is demanded in organic synthesis. In this work, a reactor was developed for on-demand production of acetylene from calcium carbide. The reactor was manufactured by FFF additive manufacturing and comprehensively tested. Compared to gaseous acetylene from a gas cylinder, the use of the reactor is safer since acetylene was generated in the necessary amounts and immediately consumed in the reaction (Figure 1b). It is important to emphasize that storing and dosing CaC_2_ is fairly simple. The use of the reactor allows the synthesis to be carried out at atmospheric pressure and without the use of high-pressure equipment. In the absence of a gas pipeline, this makes it possible to avoid the use of bulky equipment and to reduce the dimensions of the setup to the size of the reaction vessel. The practical applicability of the designed acetylene reactor was thoroughly studied. It was successfully tested in reactions of nucleophilic addition of thiols and alcohols as well as the Ni-catalyzed addition of aryl disulfides to acetylene. The use of the reactor allowed the synthesis of target vinyl derivatives, 1,4-bis(arylthio)buta-1,3-dienes and 1,2-bis(arylthio)ethenes, with good conversions/yields. The reactor is universal and allows for generating not only acetylene, but also other gases for organic synthesis. This was shown with the examples of H_2_, CO_2_, D_2_, and C_2_D_2_, which were involved in hydrogenation, carboxylation, deuteration, and deuterated vinyl derivative synthesis, respectively. The compatibility of 3D printed parts with chemical reaction mixtures was explored, and reactor stability was investigated using scanning electron microscopy. The structural integrity of both the carbon fiber-filled nylon and polypropylene reactors was rather high even under harsh conditions, where the polypropylene reactor was more durable. Amazingly, a 3D printed reactor made from plastic was found to be highly stable and was reused up to 15 times despite the highly aggressive chemical medium. This work develops a methodology for AM application in organic synthesis with gaseous reagents.

## 2. Results

### 2.1. Design of the Reactor

The idea is to create a reusable compact device for the on-demand generation of acetylene and its subsequent reaction that will solve the problems described above when using acetylene from a cylinder. For the reactor to be compact, it should fit into a reaction vessel. This reactor should have a minimum of joints, which reduces the risk of possible uncontrolled and significant leaks and the loss of generated acetylene. The possibility of varying its geometric parameters will expand the range of reaction vessels in combination with which the reactor can be used. When creating the reactor, 3D printing was used, since it allows you to quickly fabricate products with a complex structure.

**Figure 2:**
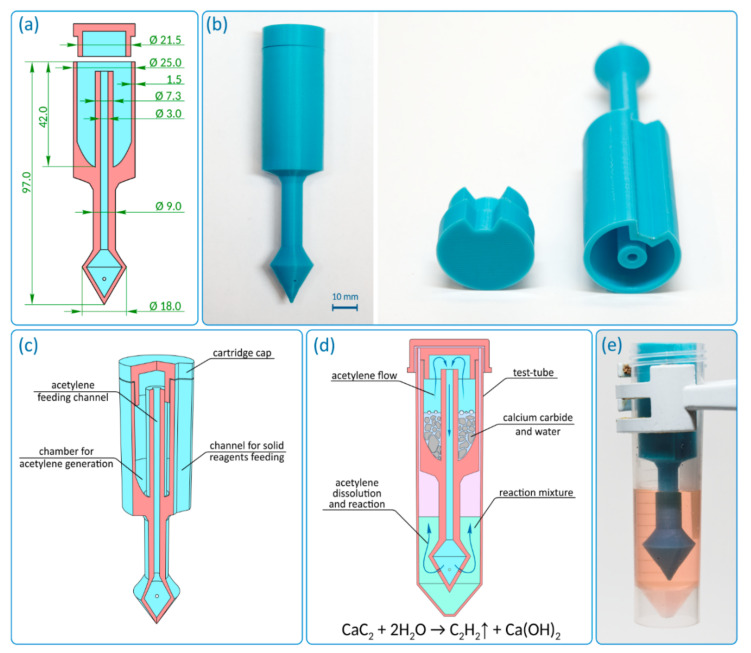
Design of reactor: (**a**) general design of the reactor, (**b**) photo of the 3D printed reactor with polylactide (PLA), (**c**) internal structure of the reactor, (**d**) working principle of the reactor, and (**e**) photo of the ready-to-use 3D printed reactor inside a tube with reaction mixture.

The reactor was designed as a hollow cylinder with a bottom, in the center of which there is a hollow tube, which subsequently extends beyond the described “barrel” (Figure 2a and Figure S1). The cylinder used has a cut out, and the total reactor height is 97 mm. The outer diameter of the cylinder is 25 mm, and the wall thickness is 1.5 mm. The inner diameter of the hollow tube is 3 mm, and the wall thickness changed from 2.15 mm inside the hollow cylinder to 3 mm outside it. The hollow tube transforms into a conical extension with a diameter of 18 mm at the end. The extension has gas exhaust holes with a diameter of 1 mm. The outer diameter of the cap is less than the inner diameter of the hollow cylinder of the reactor (21.5 mm). This is made for smooth entry of the cap into the reactor, which can be complicated in case of possible shrinkage of the material. The geometry and key dimensions of the reactor (shape, diameter, and total height) are consistent with the internal diameter and height of the reaction vessel (flask, tube, etc.). The total volume of reagents for the gas generation process defines the internal volume of the hollow cylinder and the external diameter of the central hollow tube. The length of the lower part is defined by the height of the solution in the reaction vessel and the necessary immersion depth of the reactor. The thickness of the outer wall is selected so that the wall is gas tight. The described reactor was designed for use in 50 mL polypropylene tubes. It should be noted that the geometrical parameters of the reactor and a number of the gas exhaust holes can be changed/customized if necessary. In the Supplementary Materials, we provide the computer-designed STL files, which can be used to tune the acetylene reactor for a particular vessel.

The first prototype was 3D printed with polylactide (PLA) and was monolithic (Figure 2b). The reactor contains the following functional components: an acetylene generation chamber, an acetylene feeding channel, and a cap (Figure 2c). The acetylene generation chamber is designed for the charging solid gas precursor (i.e., CaC_2_) and gas evolution reaction. The chamber is connected to the distributor (conical extension with gas exhaust holes) through the feeding channel. The acetylene generation chamber has a channel for solid reagent feeding (side cutout), which allows reagents to be loaded into the reaction vessel without removing the reactor.

First, the reagents for gas generation are loaded into the acetylene generation chamber (CaC_2_ + water/solvent). Then the chamber is immediately closed with a cap. To seal the cap and tighten the joint, silicone grease may be used. After the start of the reaction, the evolved gas passes from the chamber through the feeding channel to a distributor (Figure 2d). The distributor divides the gas flow into several individual exhausts. The holes are located at a height of ~5 mm to avoid contamination of the reaction solution in the vessel by the components from the acetylene generation chamber. When the components enter the feeding channel, they remain in the lower conical part of the reactor and do not interfere with the escape of gas through the holes. The manufactured acetylene reactor conveniently fits inside the tube and delivers acetylene directly into the reaction mixture (Figure 2e and Figure S2).

### 2.2. Evaluation of Materials for 3D Printing of the Reactor

In general, organic synthesis procedures are carried out in suitable solvents (e.g., acetone, chloroform, DMF, etc.). Parts made of plastics (e.g., 3D printed parts) may be incompatible with various solvents. To select a suitable material for reactor manufacturing, it is necessary to test the resistance of the plastics to the desired solvents.

A previous study showed that nylon, carbon fiber-filled nylon (CF-nylon), polypropylene (PP), polyethylene (PE), and polyoxymethylene (POM) are the most resistant materials to common organic solvents [74]. CF-nylon has the advantage of less shrinkage during AM than native nylon. To study a specific reaction system, we tested the resistance of three materials (PLA, CF-nylon, and PP) to acetonitrile and DMSO using an experimental procedure published earlier (Table 1) [74]. For this purpose, a 3D printed test model was placed in an empty bottle with an outer diameter of 30 mm and a height of 70 mm. On top of the object was a steel bead that served as an indicator of mechanical integrity. The vial was carefully filled with 20 mL of solvent and tightly capped. Then, the behavior of the part was observed for 20 h. The fall of the bead indicates the loss of mechanical integrity. PLA in acetonitrile undergoes swelling and subsequent delamination (detachment of the layer from one another), which leads to destruction of the part, and exposure to DMSO leads to slight swelling and transfer of the PLA dye into the solvent. CF-nylon and PP proved to be quite resistant to these solvents. There were no signs of a change in the structure of the part. For these reasons, the reactors were 3D printed with PP and CF-nylon for practical use. To obtain 3D printed reactors of high quality, the additional optimization of FFF parameters was carried out (Table S1 and Figure S3).

It should be noted that the products obtained by the FFF method are characterized by the anisotropy of mechanical properties: in the plane of the layers, the strength is higher than that between the layers. This should be taken into account when developing products for specific purposes. In some cases, a preliminary analysis of the mechanical properties of the resulting products is required [75,76,77,78]. However, in this work, reactors are developed for use in organic synthesis, in which low mechanical forces are applied. Gas generation occurs at atmospheric pressure and does not lead to an increase in pressure inside the reactor due to the immediate release of gas through the exhaust holes of the distributor.

### 2.3. Experimental Usage of the Reactor

Calcium carbide was added to the acetylene generation chamber, and then a certain amount of water was added to the chamber to initiate the formation of acetylene, followed by closing the reactor with a cap. The exhaust acetylene flows through the feeding channel and escapes through the holes at the bottom. The lower part of the reactor should be placed in a solvent to saturate the reaction mixture with acetylene. The holes distribute the released acetylene into several streams, which increases the efficiency of solvent saturation. The intensity and time of gas evolution can be controlled by changing the concentration of water added to CaC_2_, which is achieved by dilution with another solvent inert to CaC_2_ (e.g., DMF or DMSO), as well as by gradual (or portion-wise) addition of water. An illustration of the use of the reactor is shown in Figure S2.

**Figure 3:**
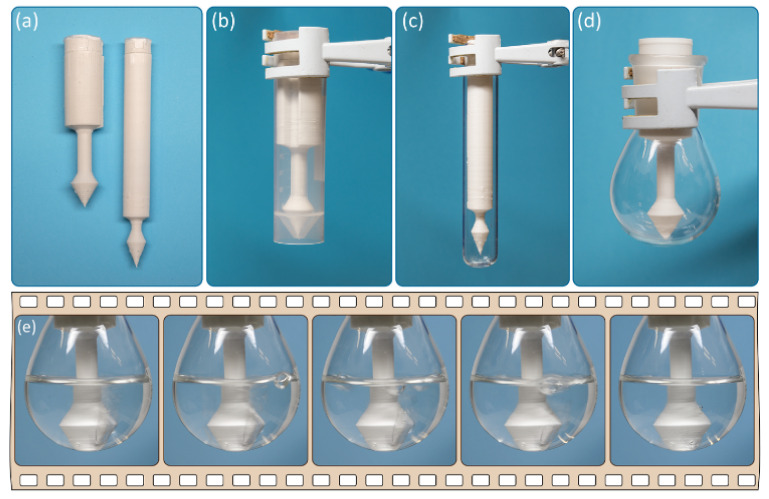
Reactors for acetylene generation 3D printed with PP: (**a**) different sizes of the reactors, (**b**) reaction setup for synthesis of 1,4-bis(arylthio)buta-1,3-dienes, (**c**) reaction setup for synthesis of 1,2-bis(arylthio)ethenes, (**d**) reaction setup for synthesis of vinyl derivatives, and (**e**) snapshots of acetylene release.

The use of computer-aided design of the reactor makes it easy to adapt the geometric parameters of the reactor to various reaction glassware (test tubes, flasks, etc.). Two different reactors with varying diameters and lengths are shown in Figure 3a.

Indeed, after customization of the geometry and 3D printing, the developed reactor was suitable for vessels of various shapes and sizes, e.g., test tubes and flasks (Figure 3b–d). After charging with calcium carbide and water, the generated acetylene gas was bubbled through the solution inside the reaction vessel (see Figure 3e).

### 2.4. Evaluation of the Reactor Efficiency and Integration of Gas Drying and Flow Meter Capabilities

A special validation was carried out to evaluate the efficiency of acetylene generation in the developed 3D printed reactor and for comparison with the standard procedure when acetylene is supplied from a cylinder (Figure 1a,b). For this, two independent saturation experiments were carried out. Acetylene was bubbled through DMSO at room temperature (~22 °C) for 20 min from two feeding sources: a standard gas cylinder and the reactor developed here (see Figure 1). Then, the concentration of acetylene in the solution was measured by ^1^H NMR spectroscopy using an internal standard. In both cases, the concentration of acetylene in DMSO was the same within the experimental accuracy: 0.52 ± 0.03 mmol/mL for the developed reactor and 0.47 ± 0.12 mmol/mL for a standard cylinder (Figure S4). The local generation of acetylene in the developed reactor resulted in a better reproducibility of the concentration of dissolved acetylene.

**Figure 4:**
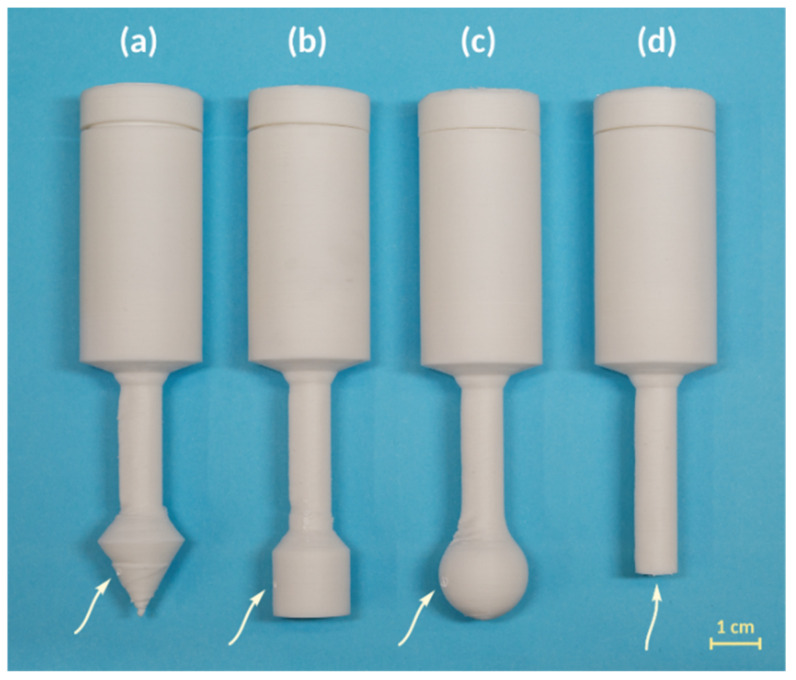
3D printed plastic reactors with distributors of different shapes (arrow points on gas exhaust holes): (**a**)—conical; (**b**)—cylindrical; (**c**)—spherical; (**d**)—without a tip.

*Varying the shape of the gas distribution tip*. A conical gas distribution tip may not fit in some cases. Additive manufacturing allows for varying the shape of this part of the reactor to better fit the bottom of the reaction vessel. We tested the distribution of gaseous acetylene depending on the shape of the lower part of the reactor. We 3D printed several types of reactors (Figure 4) with different distributors and tested them in saturation experiments. Plastic reactors were 3D printed with spherical and cylindrical distributors and without a tip (Figure 4b–d and Figure S5). Saturation experiments showed the same acetylene concentrations in all cases (Table 2). Therefore, the shape of the distributor is flexible and can vary depending on the specific reaction or equipment.

*Reactor with drying compartment*. Acetylene generated in a designed reactor may be wet due to the use of water to produce gas. Some reactions are tolerant to the presence of trace water, and acetylene can be used directly. However, the presence of water may be fatal for a variety of reactions (especially those catalyzed by metals). To integrate the drying compartment, we increased the internal diameter of the feeding channel inside the acetylene generation chamber from 3 to 5.3 mm due to the decrease of its wall thickness. Extra space inside the feeding channel was filled with the drying agent (granular anhydrous calcium chloride). The reactor was tested in saturation/drying experiments, which were similar to the saturation experiment described above. Acetylene was bubbled through DMSO at room temperature (~22 °C) for 20 min with the use of the reactor. Then, the concentration of water in the solvent was measured by Karl Fischer titration. The experiment showed the efficiency of the drying compartment (Table 3). It should be noted that the increase in water concentration during bubbling is partially caused by the adsorption of moisture from air. When necessary, the diameter or height of the drying compartment can be enlarged.

**Figure 5:**
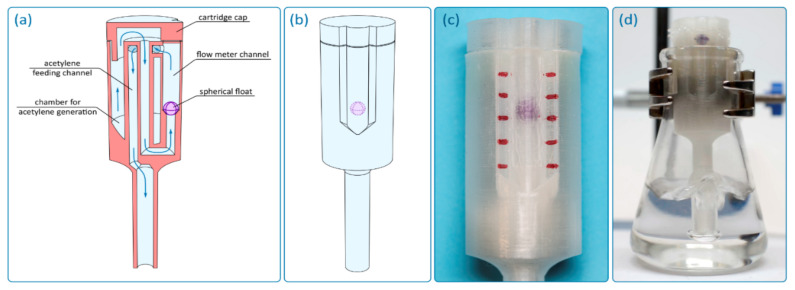
Reactor with an integrated float-type flow meter: (**a**) sectional view of the reactor, illustrating the system of channels through which the gas flow moves (shown by blue arrows); (**b**) general schematic view of the reactor; (**c**) detailed view of the reactor with the float in middle position; (**d**) during the acetylene release, the float moves to the upper part of the flow meter channel.

*Reactor with an integrated float-type flow meter*. Additive technologies make it possible to carry out significant modifications of the reactor to optimize its design in various tasks. In particular, this work demonstrated the possibility of full integration into the reactor of a float-type flow meter while retaining the possibility of using the reactor precisely as a cartridge for generating gases inside a flask or test tube (Figure 5). In a reactor with a flow meter, gas, after being generated in the chamber, enters a central channel (Figure 5a,b), which is connected to the flow meter channel. After passing through the flow meter channel, the gas enters the feeding channel and goes further into the reaction mass. Thus, to ensure the functionality of the flow meter, the gas flow changes its direction of motion several times. The flow meter channel is cylindrical, and a spherical plastic float is used as a flow indicator. To make the position of the float visible, the reactor was made of transparent plastic. It should be noted that FFF technology does not allow for obtaining completely transparent parts, even in the case of using completely transparent plastics, since the walls of the part contain many defects that scatter light. However, when using thin walls and with some optimization of FFF parameters, the transparency of the reactor is sufficient to visually identify the position of the float (Figure 5c,d). The float can be either a ready-made plastic ball of suitable size and weight or a ball made by additive manufacturing. In this case, the ball was 3D printed from two halves, which were then joined together; that is, the float was hollow to ensure optimal mass. This makes it possible to finely adjust the mass of the float by adding small pieces of plastic or glue drops to the inner cavity. The integrated flow meter can be used not only for a qualitative indication of the gas flow rate, but also for quantitative measurements since it allows for calibration with an external compressor with a precisely set gas flow rate (Figure S6).

### 2.5. Application of the Reactor in Organic Synthesis

Three synthetic procedures of practical importance were chosen to test the performance of the 3D printed reactor. We used the following procedures: synthesis of vinyl derivatives by nucleophilic addition of alcohols, thiols, and amines [79]; synthesis of 1,4-bis(arylthio)buta-1,3-dienes by nickel-catalyzed addition of aromatic disulfides to acetylene under homogeneous conditions [80]; and synthesis of 1,2-bis(arylthio)ethenes by nickel-catalyzed reaction under heterogeneous conditions [80].

It should be noted that the mode of adding a liquid reagent to a solid in a gas generation chamber is determined by the dispersity of the solid reagent and the concentration of the liquid reagent. In the case of powder, liquid should be added dropwise to avoid excessive gas evolution, which could lead to the contamination of the reaction solution with reagents from the gas generation chamber. In the case of a granular reagent, adding the liquid reagent in one portion may be sufficient for sustained gas evolution. As a general rule, the smaller the particle size of the solid reagent is, the more accurately the liquid must be fed. The intensity of the gas evolution process can be reduced by diluting the liquid reagent.

**Figure 6:**
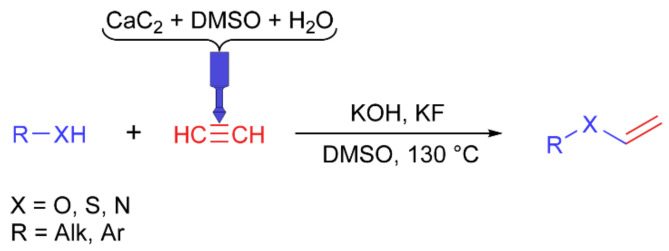
Vinylation reaction.

*Synthesis of vinyl derivatives*. CF-nylon and PP reactors were tested in the vinylation reaction (Figure 6). An additional hole was drilled in the cap and sealed with rubber to slowly supply water from a syringe to the carbide chamber. Carbide and DMSO were loaded into the acetylene generation chamber of the reactor, benzyl alcohol was used as a model substrate, and KOH and KF were loaded into the flask. Then, the calculated amount of water was injected into the carbide chamber through the septum. After optimization of the reaction conditions (Tables S2 and S3), the scope of other substrates was tested (Table 4).

The conversion was higher with the CF-nylon reactor than with the PP reactor. Surprisingly, despite the harsh reaction conditions, both 3D printed reactors were stable. Neither the superbasic medium nor the high temperature resulted in visible destruction or melting of the reactors. Compared with each other, the PP reactor was more stable than the CF-nylon reactor. The decrease in the yield of some products is associated with the high volatility of the final products.

*Synthesis of 1,4-bis(arylthio)buta-1,3-dienes*. In the reaction of aromatic disulfide addition to acetylene, the competitive formation of 1,2-bis(arylthio)ethene and 1,4-bis(arylthio)buta-1,3-diene is typically observed. High selectivity for the formation of one of the two products was achieved using a set of optimal reaction conditions, namely, a phosphine ligand and a solvent. Before the reaction, acetylene was bubbled through the solvent in a water ice bath for 30 min to saturate it. The diene synthesis was carried out under additional acetylene pressure, which was provided by connecting a gas cylinder to a test tube with reagents.

When pure water is added to calcium carbide, the reaction is extremely intense. As a result, the gas evolution time was insufficient to saturate the solvent (less than 10 min). For a less intense reaction, water (3 mL) was added to a suspension of CaC_2_ (3 g) in DMF (4 mL), which made it possible to increase the gas evolution time to ~30 min.

The reaction of 1,4-bis(arylthio)buta-1,3-diene synthesis was carried out under homogeneous conditions and was insensitive to moisture. It was found that the absence of stirring did not lead to a decrease in the product yield. Here, we used a 3D printed reactor with PP. Before the start of the reaction, the solvent in the polypropylene tube was saturated with acetylene using the designed reactor. Then the rest of the reagents were added. During the reaction, the reactor remained in the tube, which provided additional gas pressure.

**Figure 7:**
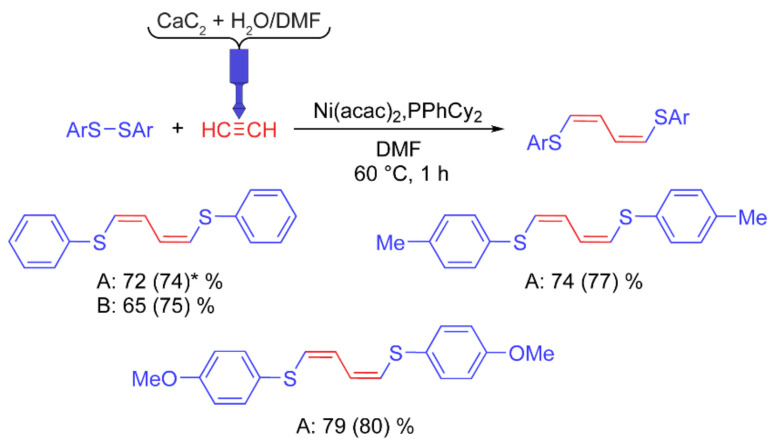
Scope of 1,4-bis(arylthio)buta-1,3-diene synthesis using the designed reactor (A) and acetylene from a gas cylinder (B). * NMR yields in parentheses.

Similar yields of 1,4-bis(phenylthio)buta-1,3-diene were obtained using the reactor developed here (Figure 7) and in the test experiment with acetylene supplied from a gas cylinder. Thus, the 3D printed reactor developed here is highly efficient and flexible for different reactions.

*Synthesis of 1,2-bis(arylthio)ethenes*. The selective formation of 1,2-bis(arylthio)ethene can be achieved with the use of heterogeneous catalysis and therefore requires stirring. Moisture is detrimental to this reaction and results in lower yields. A pre-saturated solvent is sufficient to introduce acetylene into the reaction solution. Additional acetylene pressure is not required, so the presence of the reactor in the reaction vessel during the reaction is not necessary.

Early experiments showed that the polypropylene tube was unable to withstand the pressure of acetylene, resulting in gas loss and, consequently, product traces. In the ^1^H NMR spectrum of the reaction solution, signals of water and DMF were found, which indicates that the reagents from the acetylene generation chamber ingress into the saturated solvent. This is caused by the intense release of acetylene in the first minutes after adding a DMF/water mixture (4 mL/3 mL) to CaC_2_.

**Figure 8:**
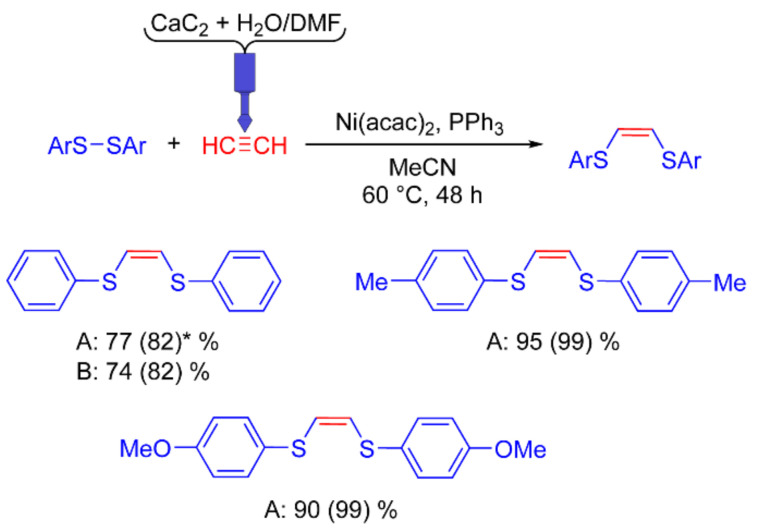
Scope of the reaction of 1,2-bis(arylthio)ethene synthesis using the designed reactor (A) and acetylene from a gas cylinder (B). * NMR yields in parentheses.

To prevent the ingress of water and DMF into the reaction solvent, the dimensions of the reactor were changed, and the height of the acetylene generation chamber was increased (Figure 3a,c). A PP reactor was used for this reaction. To avoid too intense of a reaction of CaC_2_ with water, the DMF/water mixture was added in two portions at 15 min intervals. Using this approach, together with the reaction in screw-capped tubes, yields of the target product were achieved similar to those obtained using acetylene from a gas cylinder (Figure 8).

An important advantage of using the developed reactors in comparison with gaseous acetylene is the consumed time for the experiment. The main issues of using gaseous acetylene are the availability of a cylinder with acetylene and access to the place of its storage. In a number of locations, the storage of acetylene cylinders may be restricted due to their potential explosion hazard. The use of an acetylene generation reactor saves time in preparing the experiment.

### 2.6. Generation of CO_2_, H_2_, and C_2_D_2_

Construction of the designed reactor allows it to be used to produce other gases (CO_2_, H_2_, etc.). To demonstrate this, we performed several essential organic reactions involving gases such as carboxylation of the Grignard reagent, hydrogenation of an alkene, and synthesis of deuterated products using the developed 3D printed reactor. Hydrogen and carbon dioxide are available in gas cylinders (Figure 1). However, these gases require dedicated gas transfer lines with special requirements fulfilled in each case. Our reactor is universal customized equipment, which simplifies regular laboratory experiments.

**Figure 9:**
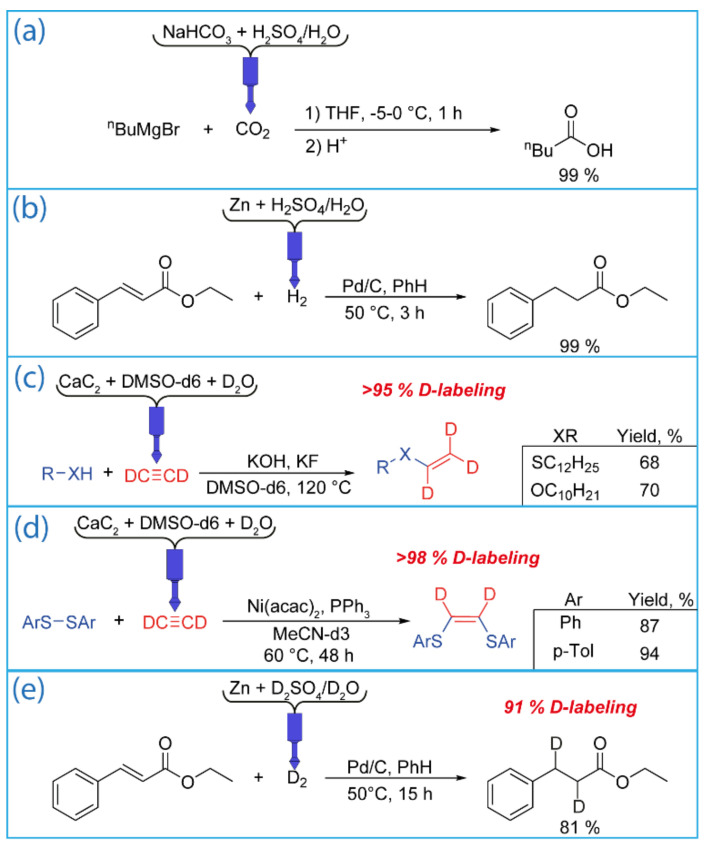
Reactions with gases generated with the use of the designed reactor: (**a**) carboxylation of n-butyl magnesium bromide, (**b**) hydrogenation of ethyl cinnamate, (**c**) nucleophilic addition to C_2_D_2_, (**d**) Ni-catalyzed addition to C_2_D_2_, and (**e**) deuteration of ethyl cinnamate.

*Carboxylation reaction (CO_2_ generation)*. The suitability of the reactor for the generation of CO_2_ was demonstrated in the carboxylation of n-butyl magnesium bromide. To generate CO_2_, sodium bicarbonate powder was loaded into a gas generation chamber. Then, an aqueous solution of sulfuric acid was added. To provide a slow and smooth generation of CO_2_, acid was added dropwise using a syringe pump for 1 h. This allowed for the valeric acid to be achieved in a high yield (Figure 9a).

*Hydrogenation reaction (H_2_ generation)*. Hydrogen was generated by adding an aqueous solution of sulfuric acid to granular Zn loaded into the gas generation chamber. The required volume of the acid was added in one portion, which provided a smooth generation of H_2_ for ~1.5 h. Hydrogenation of ethyl cinnamate was chosen as a model reaction (Figure 9b). Ethyl 3-phenyl propionate was the only product of the reaction. After 1.5 h of the reaction, the conversion of the substrate was 68%. Then, the reaction mixture was removed from the gas generation chamber, and new portions of Zn and sulfuric acid were added. The reaction continued for another 90 min. This made it possible to achieve complete conversion of the substrate.

*Synthesis of deuterated acetylene and labeled O,S-vinyl derivatives (C_2_D_2_ generation)*. The usage of gaseous reagents in laboratory practice is relatively rare due to special reaction conditions, equipment, and safety requirements. Commercially available gases in balloons can be used in some simple procedures. However, labeled gases can be packaged on demand only, shipping is extremely difficult, and special safety requirements must be fulfilled. Because of this, the price of these gases is quite high. Moreover, labeled gases are supplied under atmospheric pressure (custom synthesis), and a vacuum line is needed to retrieve the gas. These difficulties significantly complicate the reactions involving C_2_D_2_.

The use of the 3D printed reactor easily expands synthetic opportunities in the synthesis of valuable labeled gases. When the water required for the hydrolysis of calcium carbide is replaced with heavy water (deuterium oxide), deuteroacetylene C_2_D_2_ is released from CaC_2_. Compared to the literature, one can easily highlight the advantages of atom-economic D-labeling through the use of C_2_D_2_. Alternative synthetic approaches to incorporate deuterium labels are based on base- or metal-catalyzed exchange reactions [81,82,83,84,85,86], D_2_-gas use [87], or previously labeled compounds [88,89,90]. Exchange reactions consume a significant amount of deuterated starting substrate when the desired product is mixed with a many-fold excess of a deuterated source several times [91,92]. Another approach to the synthesis of deuterated compounds is the use of commercially available hydrogen gas D_2_, which may be rather expensive. The use of this approach is associated with the storage difficulties, explosive nature, limited scope of reactions, and volatility of gaseous D_2_. Thus, the scope of appropriate labeling methods is highly limited, and the synthesis of C_2_D_2_ gas without cylinders and gas equipment is an excellent opportunity.

To test a 3D printed reactor in C_2_D_2_ synthesis, a nucleophilic addition reaction of a thiol and alcohol to deuterated acetylene was carried out (Figure 9c). The same conditions were used except with D_2_O instead of H_2_O. As a result, deuterated vinyl sulfide C_12_H_25_-S-CD=CD_2_ and deuterated vinyl ether C_10_H_21_-O-CD=CD_2_ were successfully synthesized and isolated in 68%–70% yields and deuterium enrichment was greater than 95%. A similar protocol of C_2_D_2_ generation was used to conduct the reaction of Ni-catalyzed addition of Ar_2_S_2_ to deuterated acetylene (Figure 9d). This allowed for the synthesis and isolation of deuterated 1,2-bis(arylthio)ethenes with high yields (87–94%) and high deuterium enrichment (more than 98%). In both reactions, the obtained deuterated compounds were stable and treated as usual without any exchange side processes. Note that using a reactor reduces the total volume of expensive deuterated acetylene compared with the common bubbling method.

*Deuteration reaction (D_2_ generation)*. The generation of D_2_ and the subsequent deuteration reaction of ethyl cinnamate were carried out in the same manner as for H_2_ (Figure 9e). It should be noted that the deuteration reaction proceeded more slowly than the hydrogenation reaction. This is probably due to the kinetic isotope effect. After 3 h of reaction, the conversion was only 50%. Therefore, the reaction was continued for another 12 h. This made it possible to achieve a conversion of 81% with 90% deuterium enrichment. Deuterated ethyl 3-phenyl propionate was the only product of the reaction.

### 2.7. Reusability of the 3D Printed Reactor

Since the reactor was stable under the reaction conditions, we studied the possibility of reusing the reactors. The reactor was stable during operation even under the harsh conditions of the nucleophilic addition reaction. The reactor, 3D printed with CF-nylon, was able to run nine times without losing structural integrity in the nucleophilic addition of benzyl alcohol to acetylene. However, after the 10th cycle, the neck of the reactor broke off (Figure S7). The loss of the mechanical strength of the reactor is associated with the action of the solvent and reagents at high temperatures on the surface layers, which has been repeatedly shown earlier [74].

**Figure 10:**
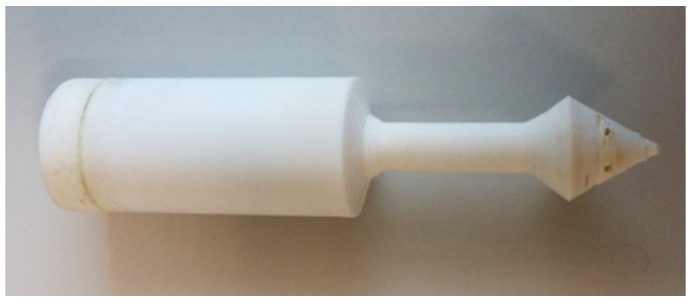
Photo of the PP reactor after the 15th reactor cycle (the reactor was used in 15 successive experiments).

In our case, the PP reactor was more stable than the CF-nylon reactor. It withstood 15 reaction cycles at 80–140 °C. No signs of destruction were observed after the 15th cycle (Figure 10 and Figure S8).

Thus, the use of reactors in other synthetic technologies seems to be very promising. CF-nylon is suitable for higher temperature processes due to the higher heat deflection temperature (HDT) of 160 °C at 0.46 MPa (T_m_ = 220 °C) in comparison with PP (HDT = 100 °C, T_m_ = 160 °C) [93].

As is currently well known, FFF parts have a porous structure and a rough surface. This can be the reason for the adsorption of the components of the reaction mixture, which is especially important in the case of catalytic processes. In some cases, even a small decrease in catalyst concentration can lead to a significant decrease in the reaction yield. Consequently, their use in catalytic reactions can be limited, in contrast to noncatalytic reactions.

### 2.8. Analysis of the Reactor Surface

**Figure 11:**
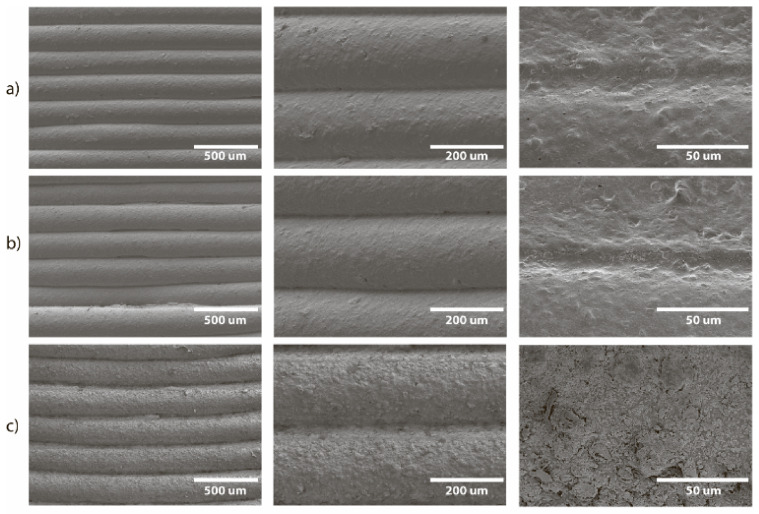
SEM images of the 3D printed reactor surface with PP at different magnifications: (**a**) before the reaction, (**b**) after one cycle of reaction of 1,4-bis(phenylthio)buta-1,3-diene synthesis (total reaction time 1 h at 60 °C), and (**c**) after five cycles of vinyl derivative synthesis (total reaction time is 5 h at 130 °C).

Harsh reaction conditions (heating up to 140 °C), organic solvents, and inorganic alkalis could completely destroy a 3D printed reactor or significantly change its structure. In addition, transparent pores and delamination may appear. To assess the consequences of the reactions with use of the reactor and the possibility of reuse, we analyzed the surface of the reactors for destruction after several cycles of reactions. The surfaces of both PP and CF-nylon reactors before and after the reactions were examined using field emission scanning electron microscopy (FE-SEM) and energy dispersive X-ray spectroscopy (EDX). The surface of the PP reactor was initially rough, with rare fine pores observed between the clearly defined layers (Figure 11a). After the reaction of 1,4-bis(phenylthio)buta-1,3-diene, no changes in the surface morphology were found (Figure 11b). EDX showed the absence of Ni, S, and P on the surface (Figure S10), i.e., no adsorption of reagents on PP occurred. In the case of the synthesis of benzyl vinyl ether, no signs of surface destruction were found after five reaction cycles, which is very promising, given the harsh reaction conditions (superbasic medium and high temperature) (Figure 11c). However, deposits of organic matter as well as KF and KOH were observed, and were confirmed by EDX (Figures S10 and S12).

**Figure 12:**
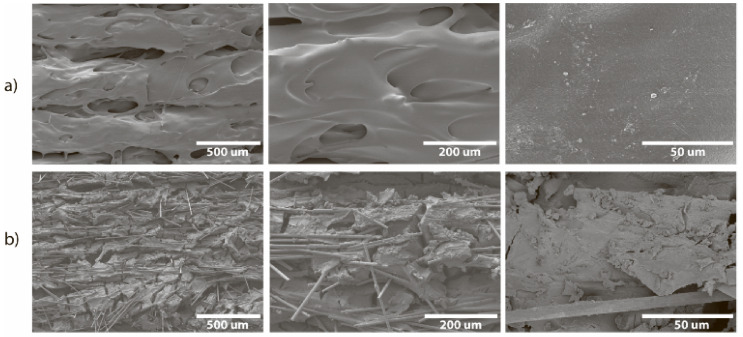
SEM images of the CF-nylon reactor surface at different magnifications: (**a**) before the reaction and (**b**) after 7 cycles of vinyl derivative synthesis (total reaction time is 11 h at 130 °C).

The surface of the CF-nylon reactor before the reaction was smooth (Figure 12a) and consisted of well-defined layers with pores between them. SEM of a cross-section prepared in liquid nitrogen revealed pores in the interior located between the layers. After nucleophilic addition, destruction of the microstructure of the reactor was found: many cracks appeared on the surface, carbon fibers emerged from the plastic (Figure 12b), and expansion of the pores inside was also observed (Figure S9). Despite this, the macrostructure of the reactor remained the same. EDX showed the presence of microcrystals containing potassium, both on the surface and inside the layer (Figures S11, S13 and S14).

## 3. Materials and Methods

*General information*. Materials used for the research were obtained from commercial sources. Polypropylene (PP, engineering grade, filled with calcium carbonate) was purchased from FL-33. Carbon fiber-reinforced nylon (CF-nylon, engineering grade, commercial name Nylforce Carbon Fiber) was purchased from FiberForce (Fiber Force Italy S.R.L., Treviso, Vicolo del Cristo, 4, Italy). The content of carbon fibers was approximately 20% by weight. All materials used for additive manufacturing were purchased in the form of filaments with diameters of 1.75 mm. Setting of FFF parameters and G-code was carried out using Simplify3D 3.1.1 software (Simplify3D, LLC, 2016). The parts were 3D printed using a Picaso 3D Designer Pro 250 printer. Reagents were obtained from commercial sources and verified by NMR prior to use. The solvents were purified according to published methods. NMR spectra were recorded on a Bruker Fourier 300 HD (Bruker BioSpin AG, Fällanden, Switzerland) at 300.1 MHz for ^1^H.

*Caution*. The experimental procedures described in the present study involve the evolution of gaseous acetylene upon the reaction of water with calcium carbide. The necessary safety requirement for experiments with gases, acetylene, and CaC_2_ should be implemented. Acetylene and hydrogen are flammable and explosive gases, which require the appropriate regulations to be implemented in all experimental work (see corresponding regulations). For all gases involved, care should be taken to avoid an accumulation of exceeding pressure. Normal gas flow should be maintained to avoid gas feeding channel blockage. The reactor should be composed of two parts, with the cap easily removed upon exceeding internal pressure (acting as a pressure release valve). Gas generation processes with the use of the developed reactor described here were optimized for an amount of solid gas precursor of approximately 1–3 g.

*3D printing of the reactors*. Reactors were created using a single AM process and were monolithic. The cap was 3D printed separately. Parameters of AM process are given in Table 5. For both materials, infill was performed with a density of 100% and a grid pattern. PP showed poor adhesion to the build platform, so 3D printing was performed on a 3 mm polypropylene sheet. In the case of CF-nylon, glue was used for better adhesion to the build platform. Reactors and caps were 3D printed in inverted position relative to Figure 2a without any additional support. After the AM process was completed, the devices were removed from the build platform using a chisel. Gas exhaust holes with diameters of 1 mm were drilled in the conical extension of the distributor. The upper edge of the acetylene generation chamber and the cap were slightly rasped for better fitting.

*Analysis of the efficiency of the reactor and drying compartment*. DMSO (15 mL) was added to a polypropylene tube. CaC_2_ grains (~2–4 mm, 3 g) were added to the acetylene generation chamber, after which a mixture of 4 mL of DMF and 3 mL of water was added in two portions at 10 min intervals. The reactor was closed with a cap (covered with silicone grease) and placed in the tube. After 20 min of gas bubbling through the solvent at room temperature, the concentration of acetylene in DMSO was determined by ^1^H NMR spectroscopy using trimethyl(phenyl)silane as an internal standard. In experiments to assess the efficiency of drying the resulting gas with a drying compartment, the concentration of water in DMSO was measured using a Mettler Toledo C10S coulometric Karl Fischer titrator.

**Figure 13:**
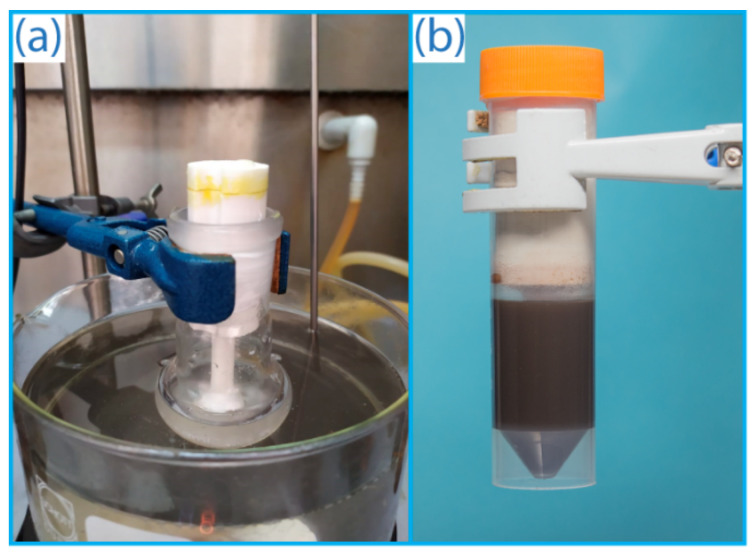
Photos of reaction setups for syntheses of (**a**) vinyl derivatives and (**b**) 1,4-bis(arylthio)buta-1,3-dienes.

*Chemical reaction: nucleophilic addition to acetylene*. DMSO (30 mL), an appropriate substrate (7.7 mmol), potassium fluoride (9.2 mmol, 537 mg), and potassium hydroxide (9.2 mmol, 518 mg) were added to a glass flask (volume 50 mL and 29 joints). Then, the reactor was placed in a flask. CaC_2_ powder (23 mmol, 1.47 g) and DMSO (2 mL) were added to the acetylene generation chamber. The reactor was closed with a cap (covered with silicone grease). The reaction mixture was stirred for 5 min and heated for the required time at the appropriate temperature (Table 4). Then, 2 mL of water was added slowly dropwise (over 5 min) through the rubber-sealed hole in the cap of the reactor using a syringe. Gas evolution was usually observed after adding 0.5–1 mL of water. Photo of reaction setup is given in Figure 13a. After the reaction, the mixture was diluted with a deuterated solvent, and the conversion was measured by ^1^H NMR.

*Chemical reaction: Ni-catalyzed addition of diaryl disulfides to acetylene leading to selective 1,4-bis(arylthio)buta-1,3-dienes formation*. The threaded neck of the polypropylene tube was wrapped with PTFE tape. The tube was flushed with argon. Ni(acac)_2_ (3 × 10^−2^ mmol, 7.7 mg) was added to the tube and dissolved in DMF (15 mL). CaC_2_ grains (~2–4 mm, 3 g) were added to the acetylene generation chamber, and then DMF (4 mL) and water (3 mL) were added. The reactor was immediately closed with a cap (covered with silicone grease) and placed in a polypropylene tube with Ni(acac)_2_ solution. The tube with the reactor was placed in a water ice bath. PPhCy_2_ (3 × 10^−1^ mmol, 82.2 mg) and Ar_2_S_2_ (1 mmol) were dissolved together in DMF (1 mL). After 20 min of bubbling acetylene, a solution of disulfide and phosphine was added to the tube, and the reactor cap was removed. The tube was closed and heated at 60 °C for 1 h. Photo of reaction setup is given in Figure 13b. No stirring was applied. The open reactor remained in the polypropylene tube during the reaction. After completion of the reaction, the solvent was evaporated on a rotary evaporator, and the product was purified by flash chromatography on silica gel 60 (0.15–0.40 mm) eluting with a hexane/CH_2_Cl_2_ gradient. Products were identified by NMR according to literature data [80]. The purity of isolated products was over 98%.

*Chemical reaction: Ni-catalyzed addition of diaryl disulfides to acetylene leading to selective 1,2-bis(arylthio)ethene formation*. The tube was flushed with argon. CaC_2_ grains (~2–4 mm, 3 g) were added to the acetylene generation chamber, followed by the addition of a mixture of DMF (2 mL) and water (1.5 mL). The reactor was immediately closed and placed in a tube with MeCN. The tube with the reactor was placed in a water ice bath. After 15 min, another portion of a mixture of DMF (2 mL) and water (3.5 mL) was added. Solid reagents including Ar_2_S_2_ (1 mmol), Ni(acac)_2_ (3 × 10^−2^ mmol, 7.7 mg) and PPh_3_ (3 × 10^−1^ mmol, 78.7 mg) were added to a screw-capped tube. After 45 min of bubbling acetylene, the reactor was removed, and pre-saturated MeCN (5 mL) was added to the solid reagents. The tube was immediately closed. The reaction was carried out at 60 °C with stirring for 48 h. After completion of the reaction, the solvent was evaporated on a rotary evaporator, and the product was purified by flash chromatography on silica gel 60 (0.15–0.40 mm) eluting with a hexane/CH_2_Cl_2_ gradient. Products were identified by NMR according to the literature data [80]. The purity of isolated products was over 98%.

*Chemical reaction: carboxylation of Grignard reagent*. A solution of n-butyl magnesium bromide in THF (0.7 mol/l, 7.5 mL) was diluted with THF (25 mL) in a two-neck flask (50 mL volume) in a stream of argon. The flask was closed with a rubber septum and placed in a water ice bath. NaHCO_3_ powder (3 g) was added to the gas generation chamber of a PP reactor without a neck (Figure 4d). The reactor was closed with a cap (treated with silicone grease) and connected to the flask via a needle. Then, an aqueous solution of sulfuric acid (25 wt. %, 6 mL) was slowly added dropwise (for 1 h) through the rubber-sealed hole using a syringe pump. The reaction was conducted under stirring. Then ice (30 g) was added, followed by dropwise addition of an aqueous solution of HCl (18 wt. %, 20 mL). The product was extracted with diethyl ether (3 × 50 mL) and dried under Na_2_SO_4_. To obtain the pure product, the solvent was removed on a rotary evaporator.

*Chemical reaction: hydrogenation of alkene*. Unreduced 5% Pd/C (267 mg), PhH (40 mL), and ethyl cinnamate (2.5 mmol, 441 mg) were added to a flask (50 mL volume with 29 joints). The reaction mixture was stirred for 5 min and heated at 50 °C. Zn grains (2 g) were added to the gas generation chamber of the PP reactor followed by a single addition of an aqueous solution of sulfuric acid (30 wt. %, 6 mL). The reactor was closed with a cap (covered with silicone grease) and placed in the flask. The reaction was conducted for 90 min under stirring and heating at the appropriate temperature. Gas evolution was continued for 1.5 h. Then, the reaction mixture was removed from the gas generation chamber, and new portions of Zn and sulfuric acid were added. The reaction was carried out for an additional 90 min. The reaction mixture was then cooled to room temperature and filtered through a pad of celite. To obtain a pure product, the solvent was removed on a rotary evaporator.

*Chemical reaction: nucleophilic addition to deuterated acetylene*. DMSO-d6 (7 mL), an appropriate substrate (1 mmol), KOH (2 mmol, 112 mg), and KF (1 mmol, 58 mg) were placed in a flask (50 mL volume with 29 joints) with a stirrer. CaC_2_ powder (1.5 g) and DMSO-d6 (2 mL) were added to the gas generation chamber of the PP reactor. The reaction mixture was heated at 120 °C for 5 min, and then 2 mL of D_2_O was slowly added dropwise (over 5 min) through the rubber-sealed hole in the cap of the reactor using a syringe. After complete addition of D_2_O, the solution in the flask was stirred at 120 °C for 2 h. Upon completion of stirring, the flask was removed from the bath and cooled to room temperature. The resulting vinyl ether was extracted from the reaction mixture with hexane (3 × 10 mL). The product was purified using column chromatography (silica gel 60 (0.015–0.040 mm), hexane as the eluent). Then, the solvent was removed under reduced pressure. Products were identified by NMR. The purity of isolated products was over 98%.

*Chemical reaction: Ni-catalyzed addition of diaryl disulfides to deuterated acetylene leading to selective 1,2-bis(arylthio)ethene formation*. The screw-capped tube was flushed with argon. Solid reagents including Ar_2_S_2_ (2 × 10^−1^ mmol), Ni(acac)_2_ (6 × 10^−3^ mmol, 1.5 mg), and PPh_3_ (6 × 10^−2^ mmol, 15.7 mg) were added to the tube. MeCN-d3 was presaturated with deuterated acetylene using the reactor. For this, CaC_2_ powder (1.5 g) was added to the acetylene generation chamber, followed by the addition of DMSO-d6 (2 mL). The reactor was closed. D_2_O (2 mL) was slowly added dropwise (over 30 min) through the rubber-sealed hole in the cap of the reactor using a syringe. After 30 min of bubbling C_2_D_2_ through MeCN-d3 in a water ice bath, pre-saturated MeCN-d3 (1 mL) was added to the solid reagents. The tube was immediately closed. The reaction was carried out at 60 °C with stirring for 48 h. After completion of the reaction, the solvent was evaporated on a rotary evaporator, and the product was purified by flash chromatography on silica gel 60 (0.15–0.40 mm) eluting with a hexane/CH_2_Cl_2_ gradient. Products were identified by NMR. The purity of isolated products was over 98%.

*Chemical reaction: deuteration of alkene*. Unreduced 5% Pd/C (267 mg), PhH (40 mL), and ethyl cinnamate (2.5 mmol, 441 mg) were added to a flask (50 mL volume with 29 joints). The reaction mixture was stirred for 5 min and heated at 50 °C. Zn grains (2 g) were added to the gas generation chamber of the PP reactor followed by a single addition of a solution of D_2_SO_4_ in D_2_O (30 wt. %, 6 mL). The reactor was closed with a cap (covered with silicone grease) and placed in the flask. The reaction was conducted for 90 min under stirring and heating at 50 °C. Then, the reaction mixture was removed from the gas generation chamber, and new portions of Zn and sulfuric acid were added. The reaction was carried out for an additional 90 min. Then, the reaction solution was removed from the gas generation chamber, filtered, and added back. The reaction was continued for another 12 h. After the reaction, the conversion and ^2^D incorporation were measured by ^1^H NMR.

*Electron microscopy SEM and EDX studies*. Plastic reactors after the reactions were washed with acetone or isopropyl alcohol. The samples were mounted on a 15 mm aluminum specimen stub and fixed by carbon double-sided adhesive tape. Metal coating with a thin film (10 nm) of a platinum/palladium alloy (80/20) was performed by using a magnetron sputtering method as described earlier [94]. The observations were carried out using a Hitachi SU8000 field-emission scanning electron microscope (FE-SEM). Images were acquired in secondary electron mode at a 2 kV accelerating voltage and at a working distance of 8–10 mm. The morphology of the samples was studied taking into account the possible influence of the metal coating on the surface. EDX-SEM studies were carried out using an X-ray spectrometer Oxford Instruments X-Max 80.

*Sample cross-section preparation for SEM and EDX*. The analyzed part of the reactor 3D printed with CF-nylon was dried at 70 °C for 2 h. Then, it was frozen in liquid nitrogen, followed by cutting with a knife immediately after being extracted from liquid nitrogen.

## 4. Conclusions

In common chemical practice, handling dangerous gases is highly complicated for several reasons. This work greatly expands the possibilities of involving gaseous reactants in chemical transformations using a 3D printed reactor. Additive manufacturing allows for the creation of reactors of various shapes to increase the efficiency of synthesis. A variety of materials for FFF printing allow for the optimal material to be chosen for specific purposes and tasks. In this work, we developed a compact reactor for the on-demand production of acetylene, H_2_, and CO_2_ that avoids contamination of the reaction solution with components of reaction of CaC_2_ with water. The absence of joints minimizes the risks of uncontrolled leakage and loss of acetylene. The reactor was 3D printed with commercially available industrial materials—polypropylene and CF-nylon. The versatility of the design of the reactor lies in the possibility of its use in combination with vessels of various shapes and sizes. The geometrical parameters of the reactor and shape of the distributor, as well as the number of gas exhaust holes, are adjustable parameters, which, if necessary, can be changed to better match the shape of the vessel. Moreover, possibilities of integrating gas-drying and flow meter capabilities were shown.

The applicability and versatility of the proposed reactor was demonstrated in the reaction of nucleophilic addition of alcohols, amines, and thiols to acetylene, as well as in reactions of Ni-catalyzed synthesis of 1,4-bis(arylthio)buta-1,3-dienes and 1,2-bis(arylthio)ethenes. In the first case, the reactor was used in combination with a flask, and in the second, in combination with a test tube. In the synthesis of 1,4-bis(arylthio)buta-1,3-dienes, the reactor remained in a test tube during the reaction to provide an additional acetylene pressure. It was shown that the use of the reactor makes it possible to carry out syntheses with the participation of acetylene in high yields.

The reusability of the reactor was demonstrated in the reaction of nucleophilic addition to acetylene. Despite the harsh conditions, the 3D printed reactor with CF-nylon withstood nine reaction cycles without loss of yield. The polypropylene reactor was more stable and was used 15 times without mechanical damage. This greatly facilitates the maintenance of long-term processes and allows for the reactors to be changed one after the other. Using SEM, it was shown that in the case of CF-nylon, the microstructure of the reactor is susceptible to destruction under such drastic conditions, while the microstructure of the PP reactor does not change.

The safety of acetylene-based synthesis using the described technology is significantly improved due to immediate acetylene consumption without acetylene accumulation under increased pressure. The solid source of acetylene, CaC_2_, is a cheap industrially produced reagent that is convenient for practical usage and easy to dose and handle.

One of the advantages of the developed reactor is its versatility for producing other gases, which is shown with examples of the formation of CO_2_ and H_2_ followed by carboxylation and hydrogenation reactions, respectively. Importantly, one of the most important applications of the reactor is in the generation of deuterated acetylene and D_2_, which enables atom-economic and cost-efficient deuterium labeling of organic molecules.

Thus, the proposed approach opens up new opportunities for many synthetic laboratories working with gaseous reagents to prepare functionalized organic molecules and further access to biologically active molecules as well as for chemical engineering and educational purposes.

## Figures and Tables

**Table 1:** Analysis of the stability of FFF parts made of various materials in organic and inorganic liquid media *.

Solvent	PLA	CF-nylon	PP
Water	+	+	+
Ethanol	+	+	+
Acetone	−	+	+
Acetonitrile	−	+	+
DMF	−	+	+
DMSO	±	+	+

* (+) material is stable during the experimental time, i.e., the shape of the part does not change; there is no dissolution of the outer layers of the material; (−) the material is unstable during the experiment, there is a change in shape or destruction of the part; (±) the material is moderately stable during the experimental time: swelling or slight dissolution of the outer layers is observed, the shape of the part does not change.

**Table 2:** Concentrations of acetylene in DMSO depending on feeding shape.

Reactor	Figure 4a	Figure 4b	Figure 4c	Figure 4d	Acetylene from Gas Cylinder
C (C_2_H_2_), mmol/mL	0.54 ± 0.01	0.56 ± 0.02	0.56 ± 0.04	0.55 ± 0.05	0.47 ± 0.12

**Table 3:** Content of water in DMSO before and after saturation with acetylene.

Drying Agent	DMSO before Saturation	No Drying Agents	CaCl_2_	Acetylene from Gas Cylinder
C (H_2_O), wt %	0.235 ± 0.008	0.442 ± 0.009	0.354 ± 0.003	0.335 ± 0.012

**Table 4:** Reaction conditions and product yields of the synthesized vinyl derivatives *^a^*.

№	Substrate	Reactor	Weight of CaC_2_, g	T, °C	Reaction Time, h	Conversion, Mass %
1	Benzyl alcohol	CF-nylon	1.47	130	1	99.2
		PP	1.47	120	3	92.2
		PP	1.47	140	1	99.9
2	Octanol-1	CF-nylon	1.47	130	1	54.1
3	Undecanol-1	PP	1.47	130	1	57.6
		CF-nylon	2.00	130	1	57.0
		PP	2.00	130	1	49.5
4	Citronellol	PP	1.47	130	1	67.4
		PP	2.00	130	1	69.6
		CF-nylon	2.00	130	1	62.2
5	Menthol	CF-nylon	1.47	130	1	21.8
6	Fenchol	PP	1.47	130	1	40.7
7	1,10-Decanediol *^b^*	CF-nylon	1.47	130	1	68.1
		PP	1.47	130	1	66.7
		CF-nylon	2.00	130	1	78.8
		PP	2.00	130	1	74.5
8	1-Dodecanethiol	CF-nylon	1.47	130	1	99.9
		PP	1.47	130	1	99.9
9	Thiophenol	CF-nylon	1.47	130	1	56.0
		CF-nylon	2.00	130	1	45.2
		PP	2.00	130	1	46.7
10	p-Tolyl thiophenol	CF-nylon	1.47	130	1	52.1
11	Diphenyl amine	CF-nylon	2.00	130	2	70.4
		PP	2.00	130	2	76.0
12	Indole	CF-nylon	2.00	130	1	48.1
		CF-nylon	1.47	140	1	41.1
		PP	1.47	140	1	36.0
13	Carbazole	CF-nylon	1.47	130	1	19.3

*^a^* Reaction conditions: substrate (7.7 mmol), potassium fluoride (9.2 mmol), potassium hydroxide (9.2 mmol), calcium carbide (23 mmol), and DMSO (2 mL). *^b^* 3.85 mmol of the substrate was used instead.

**Table 5:** Parameters of FFF process.

Material	Diameter of Nozzle, mm	Temperature of Build Platform, °C	Temperature of Nozzle, °C	Cooling Intensity, %	Extrusion Multiplier	Layer Height, mm
PP	0.3	80	235	20	0.98	0.20
CF-Nylon	0.5	100	245	60	0.80	0.35

## Data Availability

Data are contained in the article and Supplementary Materials.

## Supplementary Materials

Supplementary materials can be found at https://www.mdpi.com/article/10.3390/ijms22189919/s1.
