# Supplementary material for: 3D Printing to Increase the Flexibility of the Chemical Synthesis of Biologically Active Molecules: Design of On-Demand Gas Generation Reactors
Source: Int J Mol Sci. 2021 Sep 14;22(18):9919. doi: 10.3390/ijms22189919 (PMC8472564; doi:10.3390/ijms22189919)
Supplement: Supplementary file 1 [file ijms-22-09919-s001.zip › New SI/ijms-1368463-supplementary.pdf]

3D printing to increase the flexibility of the chemical synthesis of biologically active molecules: design of on-demand gas generation reactors

Kirill S. Erokhin,[1] Evgeniy G. Gordeev,[1] Dmitriy E. Samoylenko,[2] Konstantin S. Rodygin,[2] Valentine P. Ananikov [1,2]*

[1] *N. D. Zelinsky Institute of Organic Chemistry Russian Academy of Sciences, Leninsky prospect 47, Moscow, 119991, Russia*

[2] *Institute of Chemistry, Saint Petersburg State University, Universitetsky prospect 26, Peterhof, 198504, Russia.*

**E-mail: val@ioc.ac.ru*

Table of content

1. Design of the reactor....2
2. Experimental usage of reactor....3
3. Optimization of parameters of 3D printing with CF-nylon....4
4. Acetylene solubilization in DMSO....6
5. Variation of shape of lower part of the reactor....7
6. Reactor with an integrated float-type flow meter....8
7. Model reaction of vinylation of benzyl alcohol....9
8. Evolution of the reused reactors....10
9. SEM of cross-section of CF-nylon reactor....12
10. EDX spectra....13
11. EDX mapping of reactors after nucleophilic addition reaction....14

1. Design of the reactor

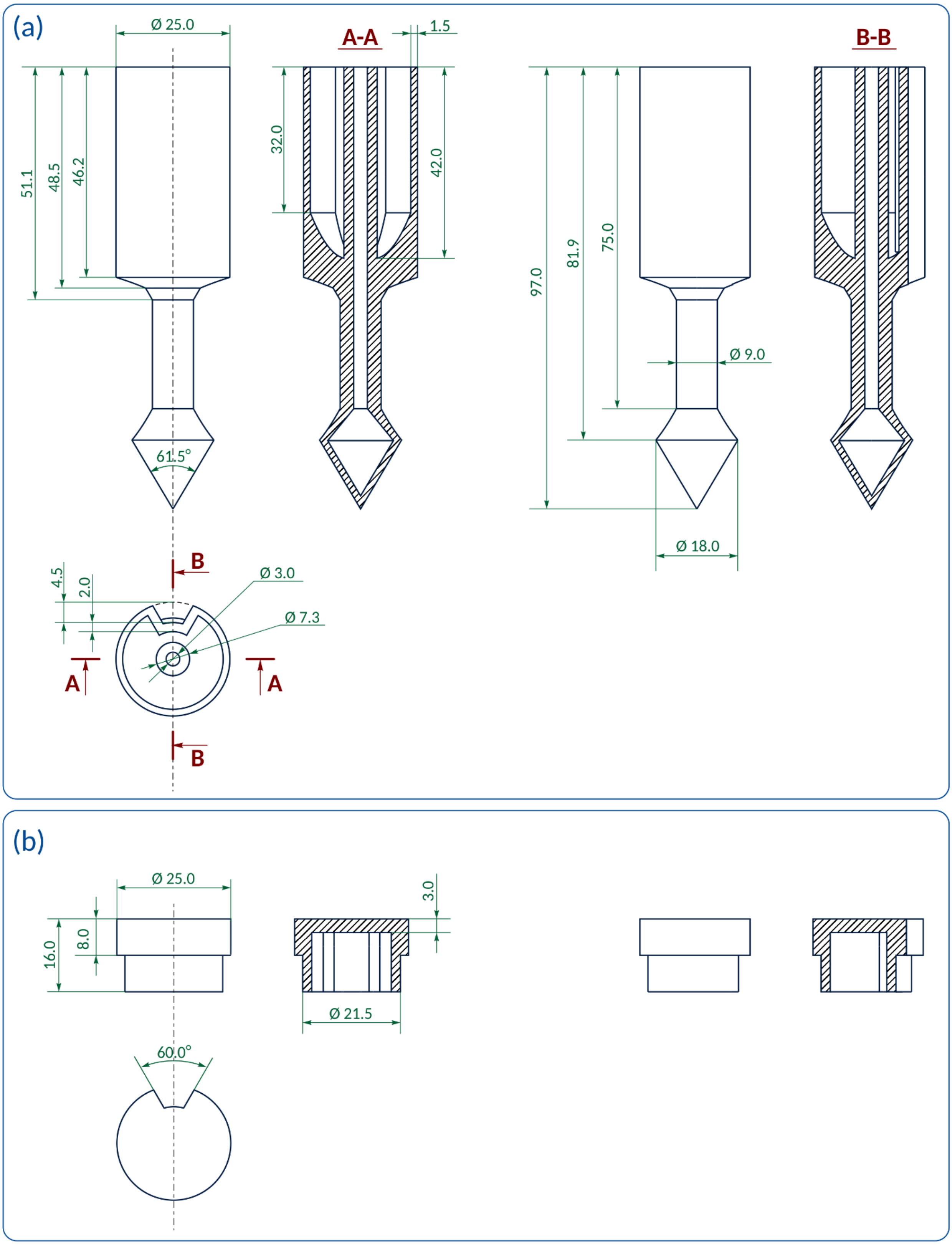

Figure S1. Draft of the reactor: a) main body; b) cap.

2. Experimental usage of reactor

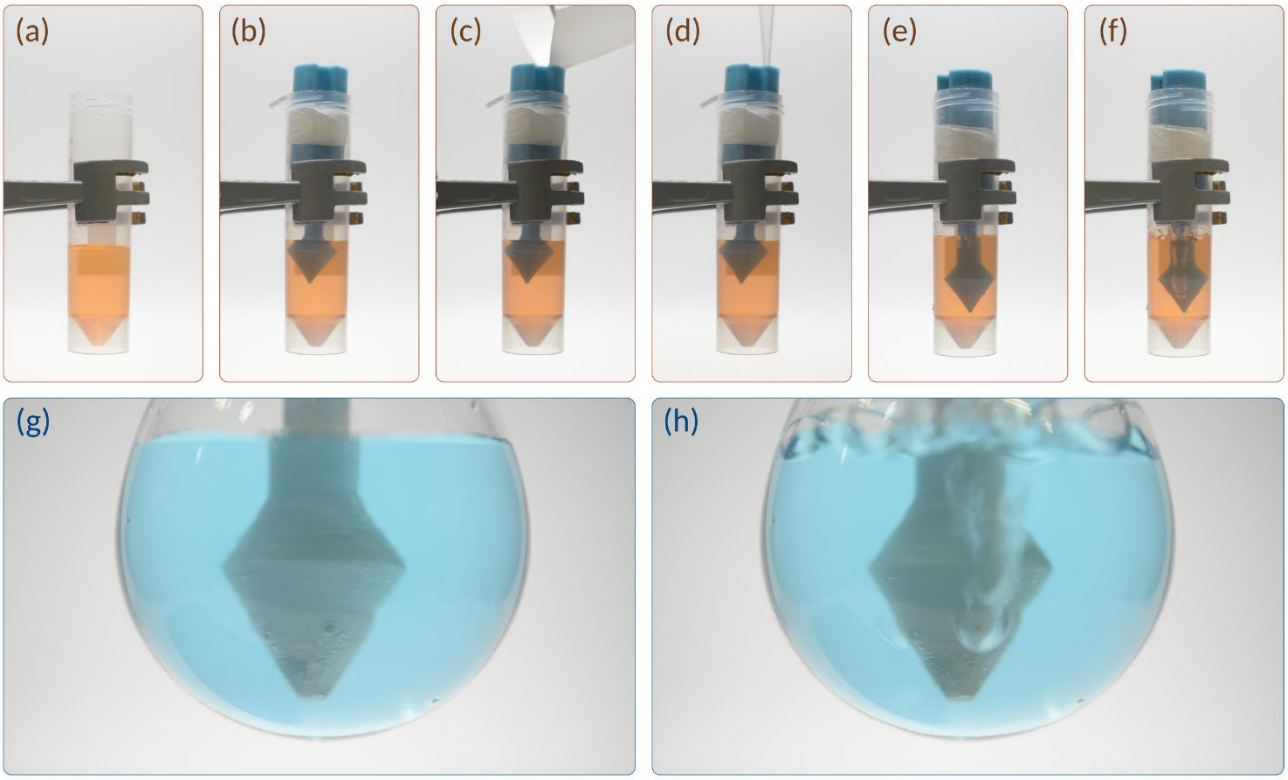

Figure S2. *Method of using a carbide cartridge for acetylene generation: (a) - initial reaction mass; (b) - inserting the cartridge into the test tube (a fluoroplastic sealant can be used to fix the cartridge); (c) adding calcium carbide to the chamber for acetylene generation; (d) adding water to the chamber for acetylene generation using a pipette (a mixture of water and an organic solvent can be used to control the rate of outgassing); (e) - sealing the cartridge with a ca and immersing the gas distributor in the reaction mass; (f) - active release of acetylene into the reaction mass; (g) - enlarged view of the gas distributor inside the reaction mass in a pear-shaped flask; (h) - an enlarged view of the gas distributor inside the reaction mass during the active release of acetylene; it is clearly seen that even with active gas releasing, the solution is not contaminated with solid particles.*

3. Optimization of parameters of 3D printing with CF-nylon

Optimization of 3D printing parameters of the reactor

The main parameters of 3D printing are the extrusion multiplier, extrusion width, and 3D printing temperature. The extrusion multiplier determines the volumetric flow rate of molten plastic during FDM 3D printing. The extrusion width corresponds to the spreading width over the underlying layer of plastic melt. The 3D printing temperature is the temperature of the heating element in the hot end where the thermoplastic filament transitions into the molten state before extrusion.

Initially, an attempt was made to 3D print a CF-nylon reactor using the previously described set of parameters to 3D print models for a chemical resistance test (Table S1, entry 1) [1]. This attempt was unsuccessful because too much material came out of the nozzle. This was fatal to the bottom of the acetylene generation chamber and made the subsequent FDM process impossible (Fig. S1). Due to the large amount of material supplied and the lack of cooling, the molten nylon did not have time to harden before the next layer was applied. Decreasing the 3D print temperature, extrusion multiplier and extrusion width and increasing the applied layer height did not improve the results (entries 2-5). Cooling the model at maximum intensity allowed the target 3D print to be completed. At 225 °C, the 3D printed prototype of the reactor was found to be rather weak, especially for the feeding channel inside the acetylene generation chamber. This is probably due to poor interlayer adhesion. In addition, the reactors were permeable: water added to the acetylene generation chamber quickly left the reactor through the pores in the walls. Raising the nozzle temperature to 245 °C led to a significant improvement in the quality of the models. However, this did not give reactor impermeability (entry 11). Increasing the extrusion multiplier to 0.9 solved this problem, but in this case, too much material came out of the nozzle, which affected the geometry of the target model and reduced its quality. Finally, the change in the cooling intensity showed that using the value of 60% allows the model to be 3D printed with high quality (entry 18).

In the case of 3D printing the reactor with polypropylene, the previously optimized set of parameters proved to be suitable for making high-quality models [1]. The extrusion multiplier was increased to 0.98 to obtain impermeable models. This did not lead to a decrease in the 3D print quality of the reactors.

---

[1] Erokhin, K.S.; Gordeev, E.G.; Ananikov, V.P. Revealing interactions of layered polymeric materials at solid-liquid interface for building solvent compatibility charts for 3D printing applications. Sci. Rep. 2019, 9, 20177, doi:10.1038/s41598-019-56350-w.

Table S1. Optimization of parameters for 3D printing with CF-nylon material.

№	3D printing T, °C	Extrusion multiplier	Cooling intensity, %	Extrusion width, mm	Layer height, mm	3D printing quality[a]	Impermeability of reactor walls
**1**	245	0,98	0	0,6	0,25	low	not printed[b]
**2**	245	0,98	0	0,6	0,35	low	not printed
**3**	235	0,98	0	0,5	0,35	low	not printed
**4**	235	0,90	0	0,5	0,45	low	not printed
**5**	225	0,80	0	0,5	0,45	low	not printed
**6**	225	0,80	30	0,5	0,45	medium	poor
**7**	225	0,80	100	0,5	0,45	medium	poor
**8**	225	0,60	100	0,5	0,45	medium	poor
**9**	225	0,70	100	0,5	0,45	medium	poor
**10**	225	0,70	100	0,5	0,35	medium	poor
**11**	245	0,70	100	0,6	0,35	high	poor
**12**	245	0,75	100	0,6	0,35	high	poor
**13**	245	0,78	100	0,6	0,35	high	poor
**14**	245	0,80	100	0,6	0,35	high	poor
**15**	245	0,90	100	0,6	0,35	medium	excellent
**16**	245	0,85	100	0,6	0,35	medium	excellent
**17**	245	0,80	20	0,6	0,35	medium	excellent
**18**	245	0,80	60	0,6	0,35	high	excellent

[a] – 3D printing quality implies coincidence of geometric parameters of the model with designed parameters, absence of visible defects, and physical integrity in the experiment.

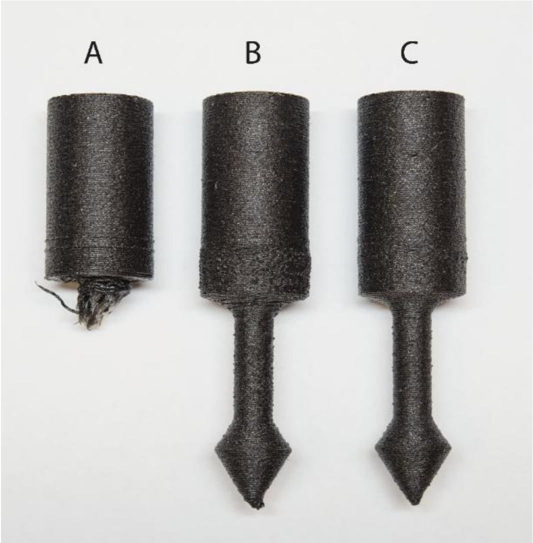

Figure S3. *Photos of models 3D printed at different set of parameters resulted in bad (a), moderate (b) and good (c) quality of 3D printing.*

4. Acetylene solubilization in DMSO

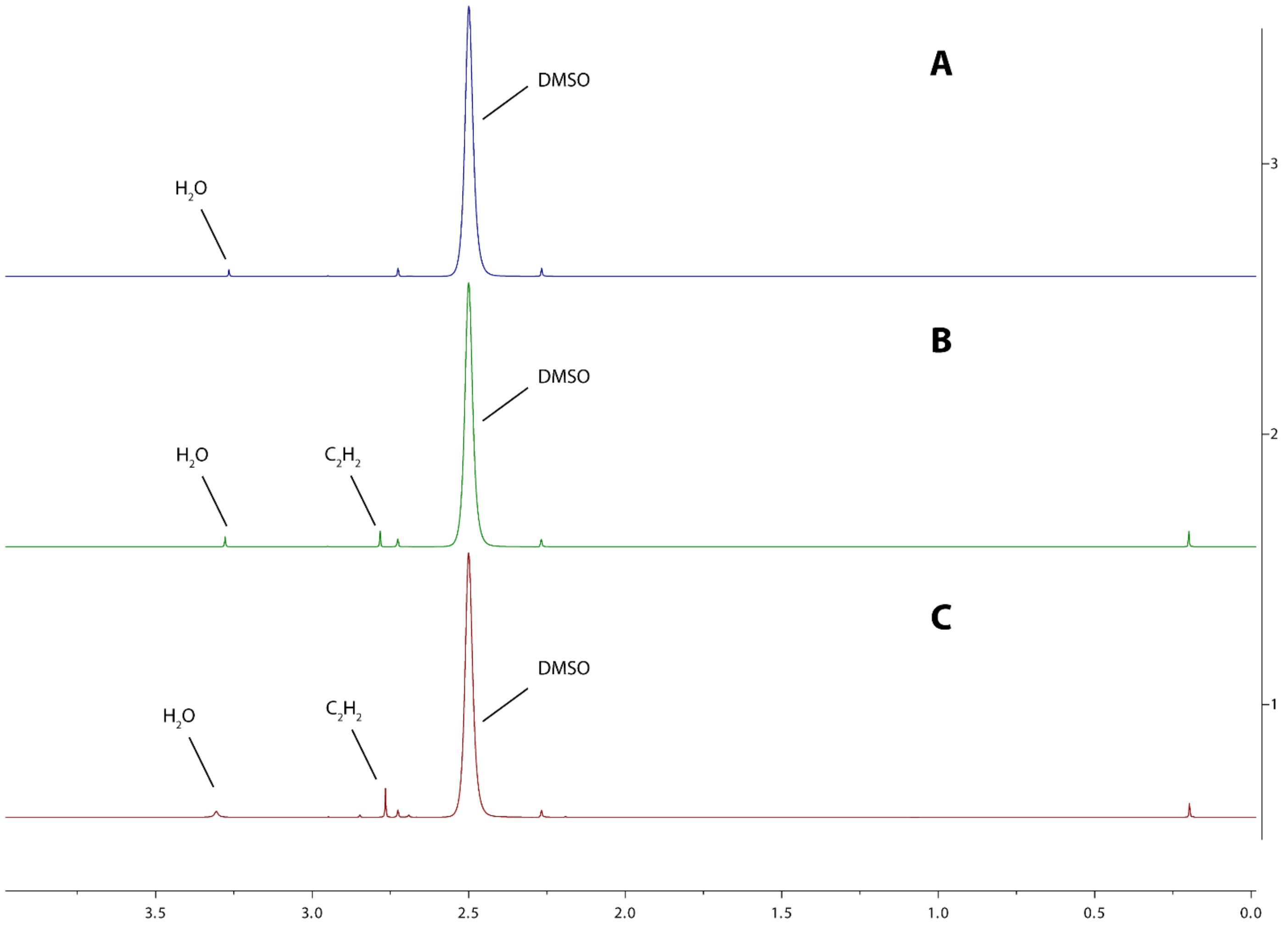

Figure S4. *^{1}H NMR spectra of DMSO-H6: a)pure; b) saturated with acetylene from gas cylinder; c) saturated with acetylene with use of developed reactor.*

5. Variation of shape of lower part of the reactor

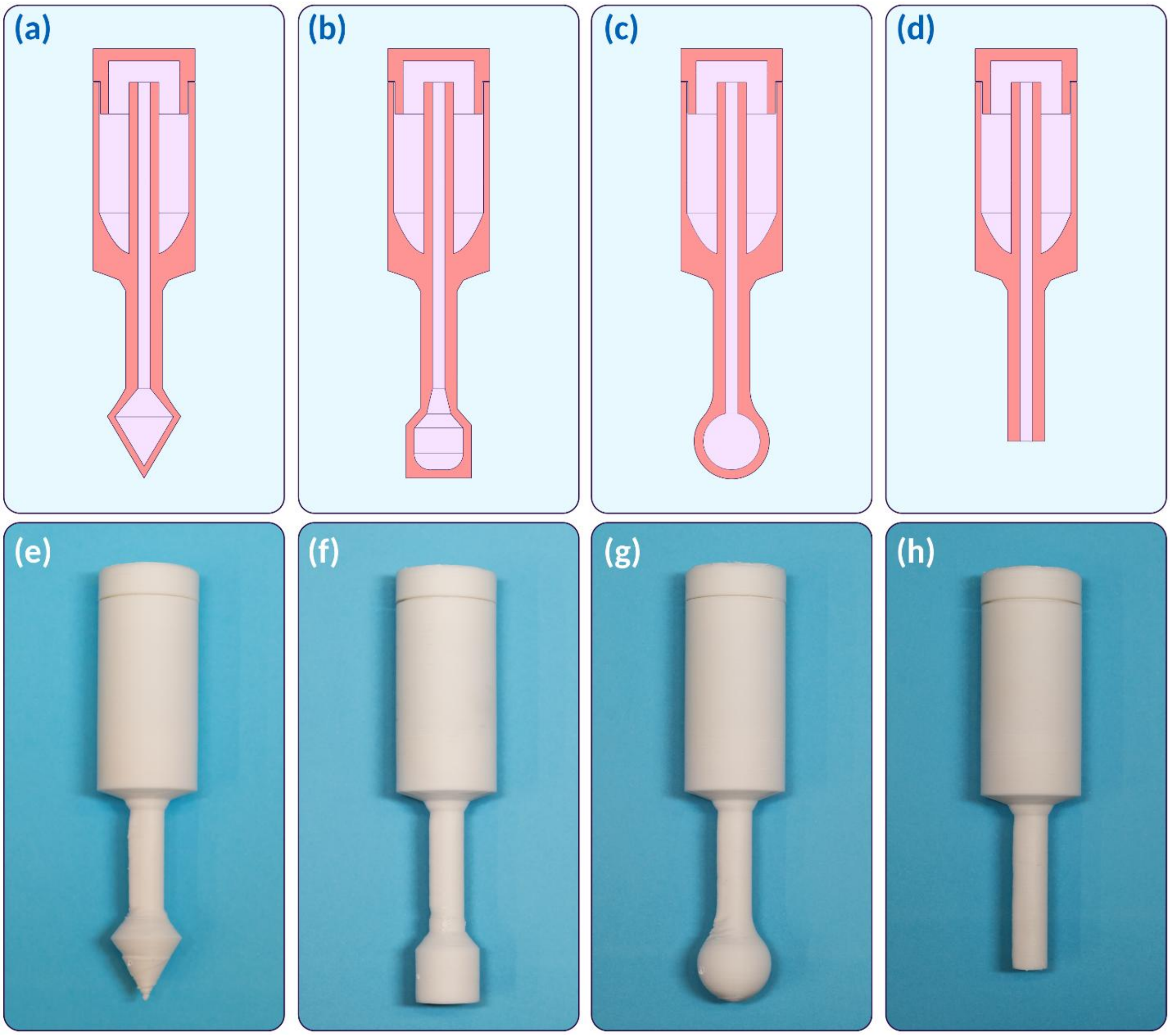

Figure S5. *Reactors with distributors of different shapes: a-d – schemes, e-h – photos.*

Shape distributor and number of gas exhaust holes are customizable and can be modified. It should be noted that manufacturing of holes with small diameter (ca. 1 mm) is easier with regular tools (e.g. drilling) while 3D printing is more proper for the holes with longer diameter.

6. Reactor with an integrated float-type flow meter

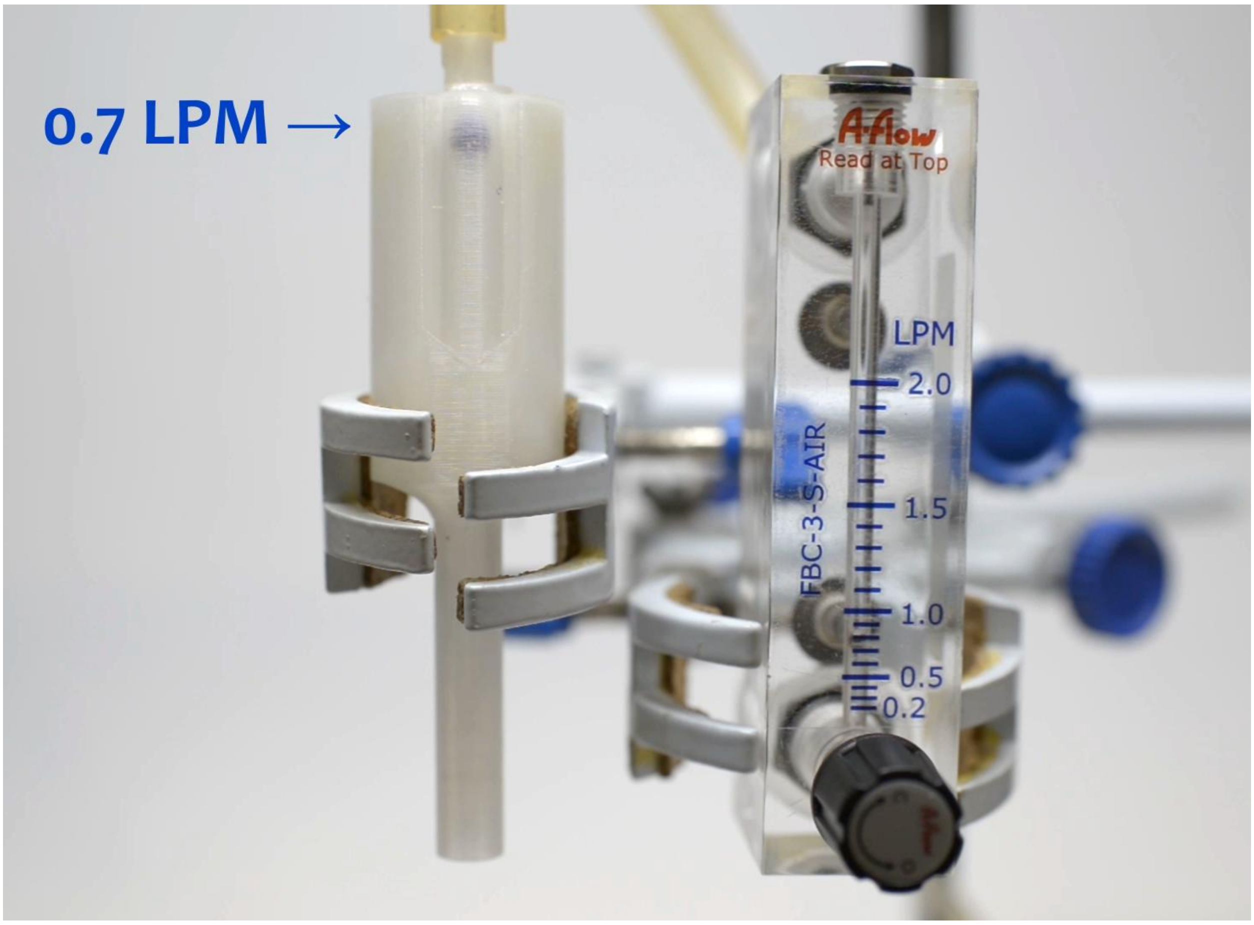

Figure S6. *Photo of calibration of integrated float-type flow meter.*

7. Model reaction of vinylation of benzyl alcohol

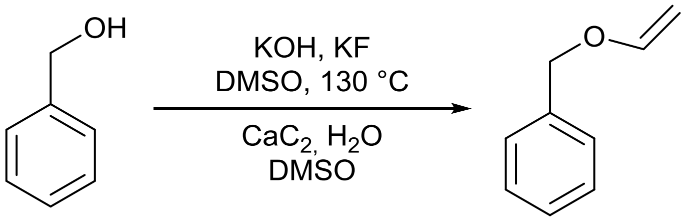

Table S2. *Optimization of reaction conditions of nucleophile addition of benzyl alcohol to acetylene (CF-nylon reactor was used).*

№	CaC_2, g	Time	DMSO, ml	H_2O, ml	Yield*, %
1	1.47	3 h	2	2	92.4
2	1.47	2 h	2	2	92.4
**3**	**1.47**	**1 h**	**2**	**2**	**96.5**
4	1.47	0.5 h	2	2	85.4
5	1.47	5 min (time of bubbling of acetylene)	2	2	71.1
6	1	1 h	2	2	87.1
7	0.5	1 h	2	2	63.6
8	1.47	1 h	3	2	88.2
9	1.47	1 h	1	2	89.7
10	1.47	1 h	-	2	95.1
11	1.47	1 h	2	1	91.7
12	1.47	1 h	2	3	89.5

Table S3. *Optimization of reaction conditions of nucleophile addition of benzyl alcohol to acetylene (PP reactor was used).*

№	Temperature, °C	Time, h	Conversion of alcohol, mass %
1	80	1	36.2
2		2	40.1
3		3	43.2
4		4	45.9
5		5	49.2
6	90	1	50.0
7		2	57.0
8		3	64.7
9	100	1	60.8
10		2	66.0
11		3	71.7
12	110	1	86.4
13		2	90.8
14		3	90.8
15	**120**	1	87.3
16		2	90.8
**17**		**3**	**92.2**
18	130	1	81.3
19		2	83.9
20		3	86.4

8. Evolution of the reused reactors

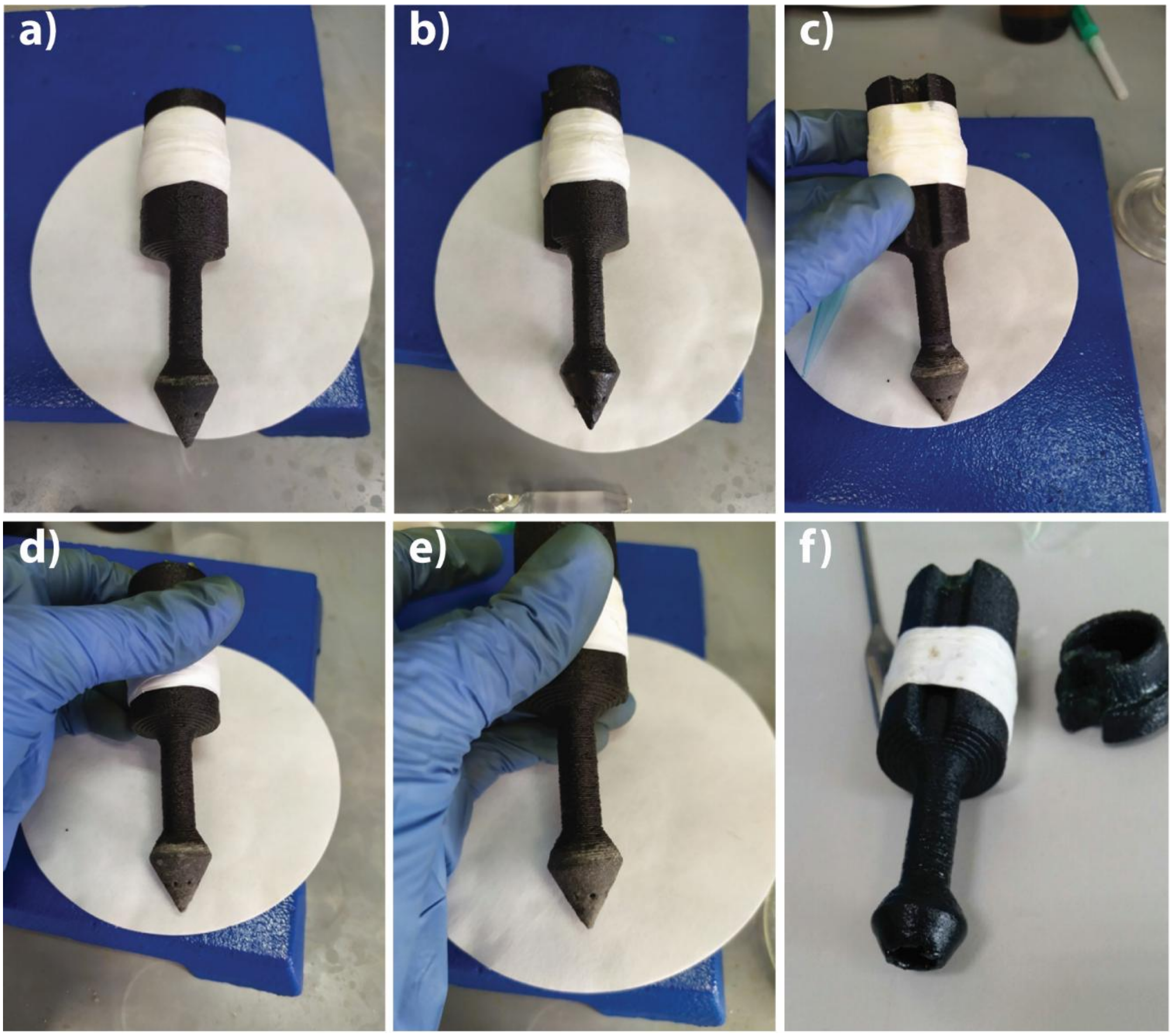

Figure S7. *Snapshots of the CF-nylon reactor after 1'st (a), 3'rd (b), 5'th (c), 7'th (d), 9'th (e), and 10'th (f) cycles of nucleophilic addition reaction.*

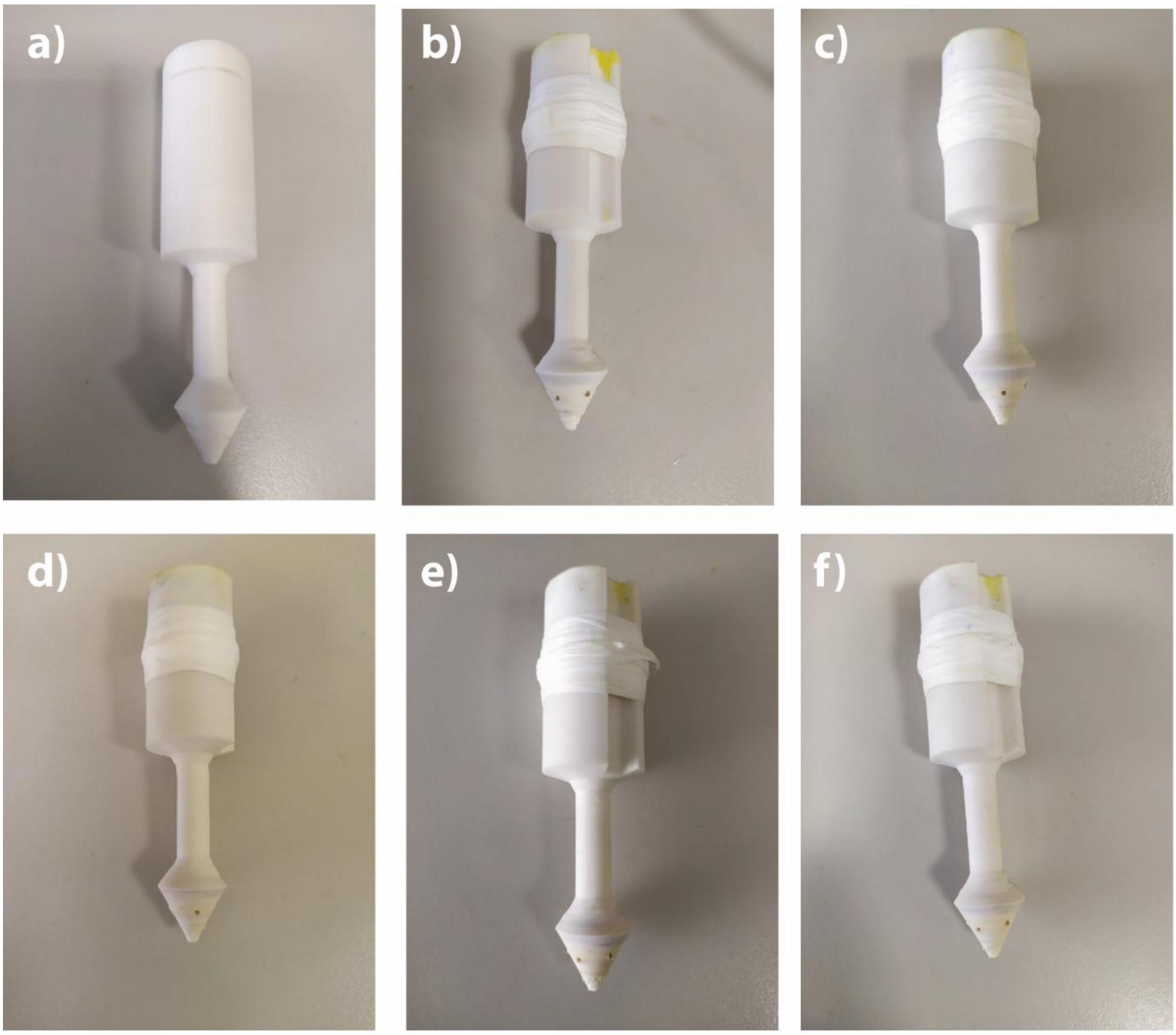

Figure S8. *Snapshots of new PP reactor (a) and after 2'nd (b), 3'rd (c), 4'th (d), 5'th (e), and 6'th (f) cycles of nucleophilic addition reaction.*

9. SEM of cross-section of CF-nylon reactor

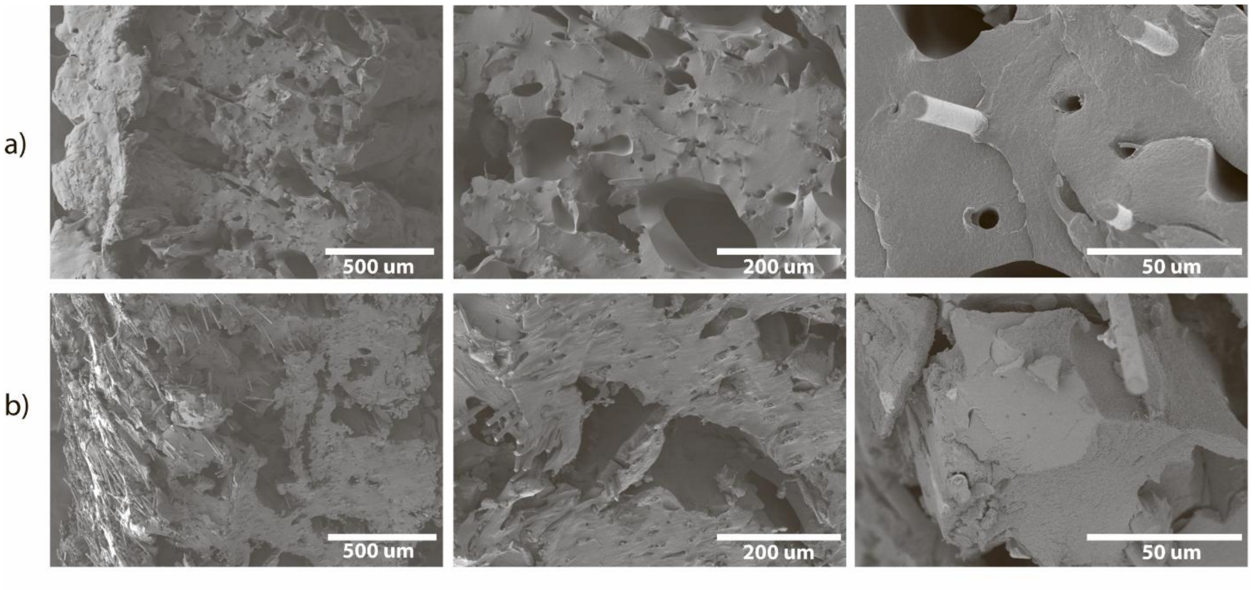

Figure S9. *SEM images of the reactor cross-section 3D printed with CF-nylon at different magnifications: a) before the reaction; b) after 5 cycles of reaction of vinyl benzyl ether synthesis (total time of exploitation is 5 h at 130 °C)*

10. EDX spectra

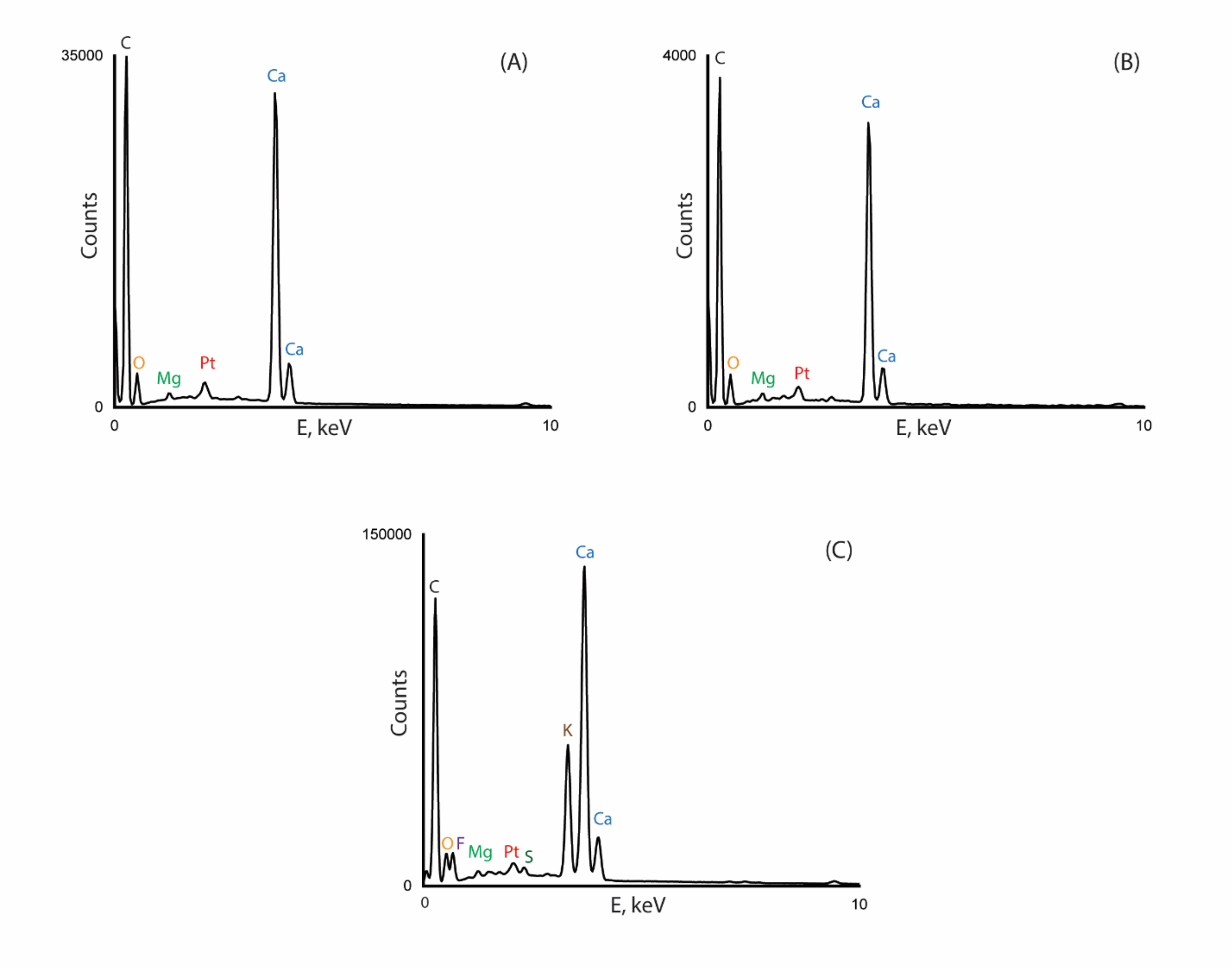

Figure S10. *EDX spectra of the reactor surface 3D printed with PP: A) before the reaction; B) after 1 cycle of reaction of 1,4-bis(phenylthio)butadiene-1,3 synthesis (total reaction time is 1 h at 60 °C); c) after 5 cycles of reaction of vinyl derivative synthesis (total reaction time is 5 h at 130 °C)*

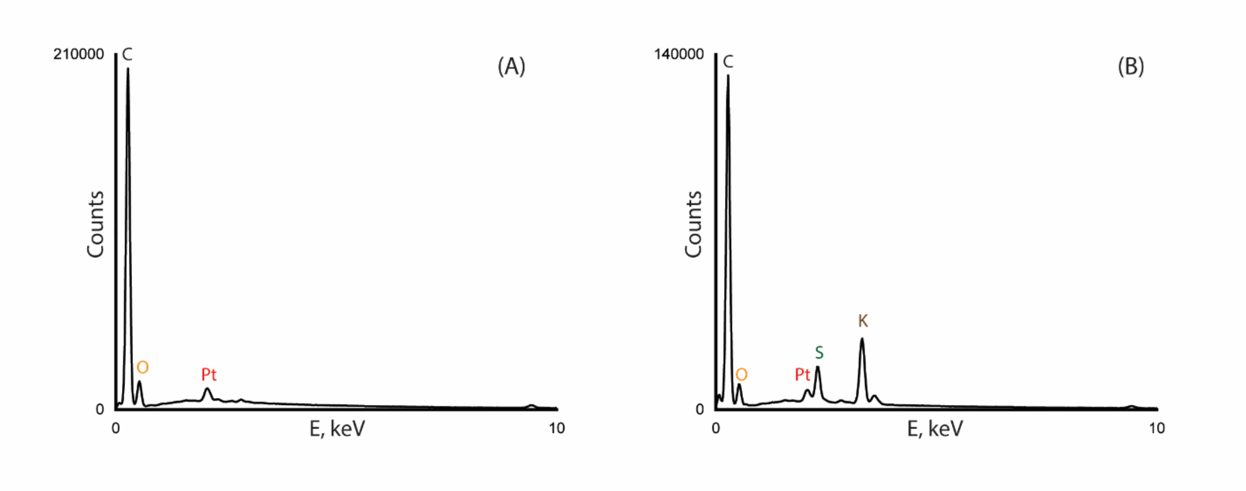

Figure S11. *EDX spectra of the reactor surface 3D printed with CF-Nylon: A) before the reaction; B) after 7 cycles of reaction of vinyl derivative synthesis (total reaction time is 11 h at 130 °C)*

11. EDX mapping of reactors after nucleophilic addition reaction

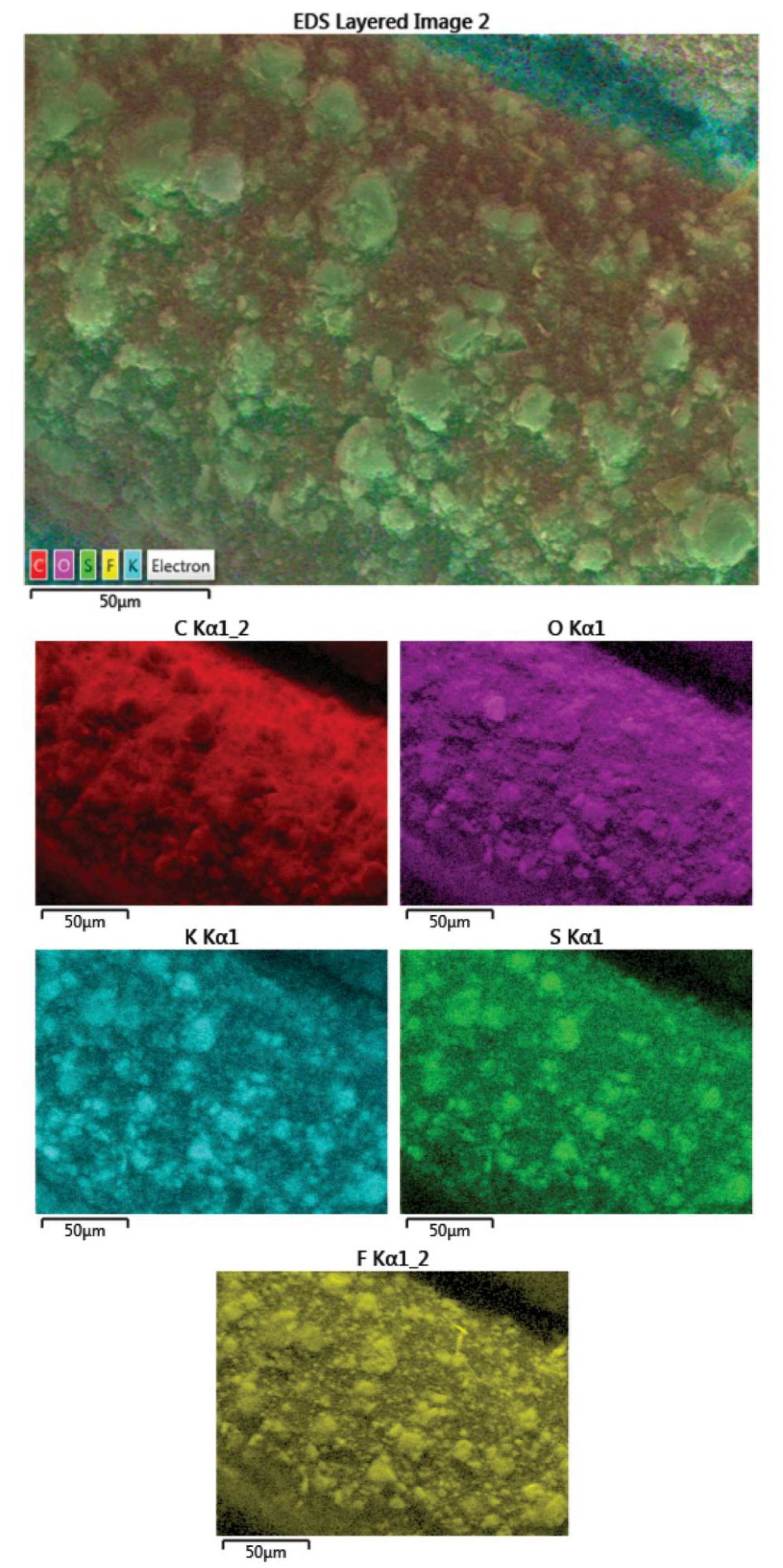

Figure S12. *EDX mapping of the reactor surface 3D printed with PP after 7 cycles of reaction of vinyl benzyl ether synthesis after 5 cycles of vinyl derivative synthesis (total reaction time is 5 h at 130 °C)*

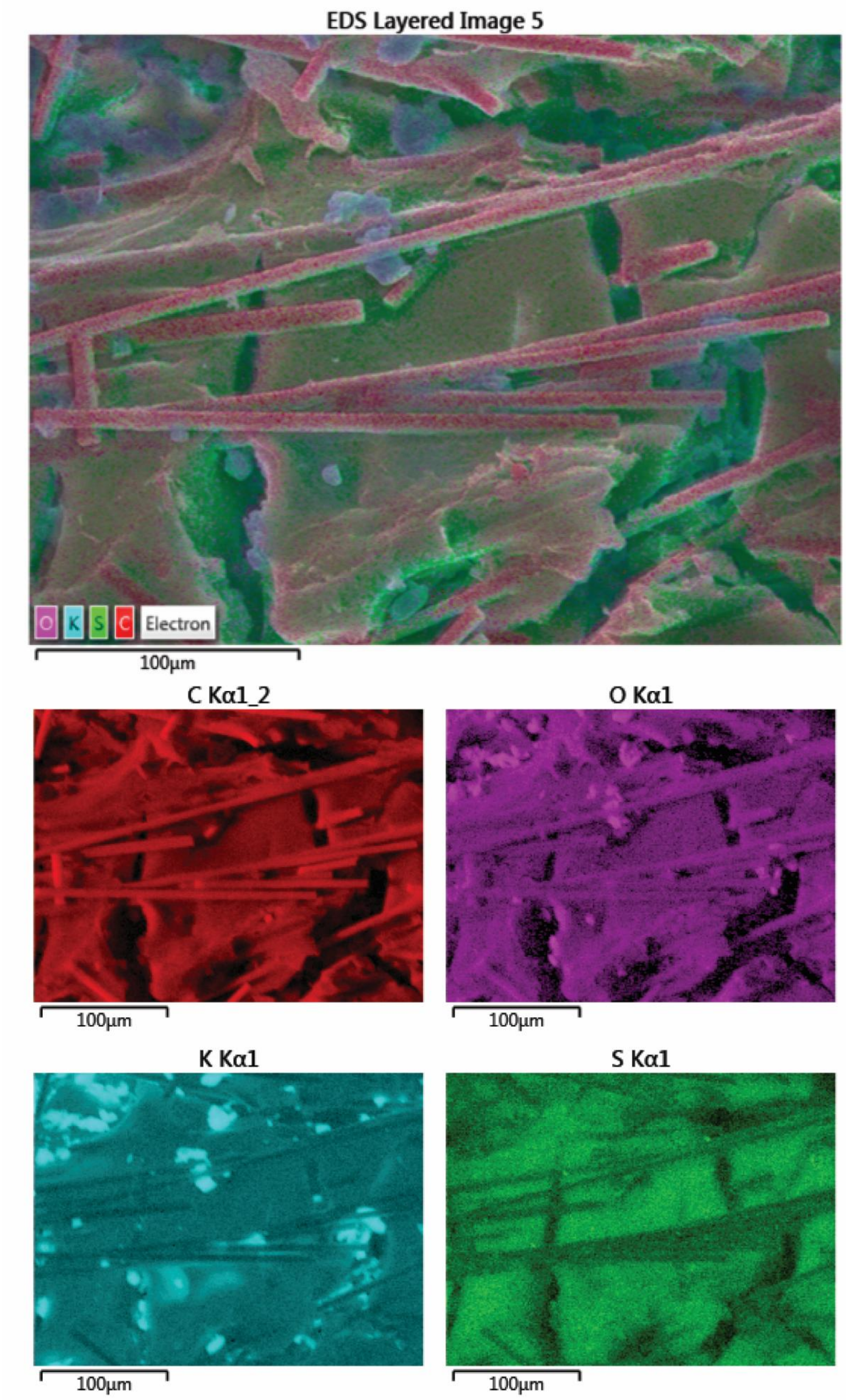

Figure S13. *EDX mapping of the reactor surface 3D printed with CF-nylon after 7 cycles of reaction of vinyl derivative synthesis (total reaction time is 11 h at 130 °C)*

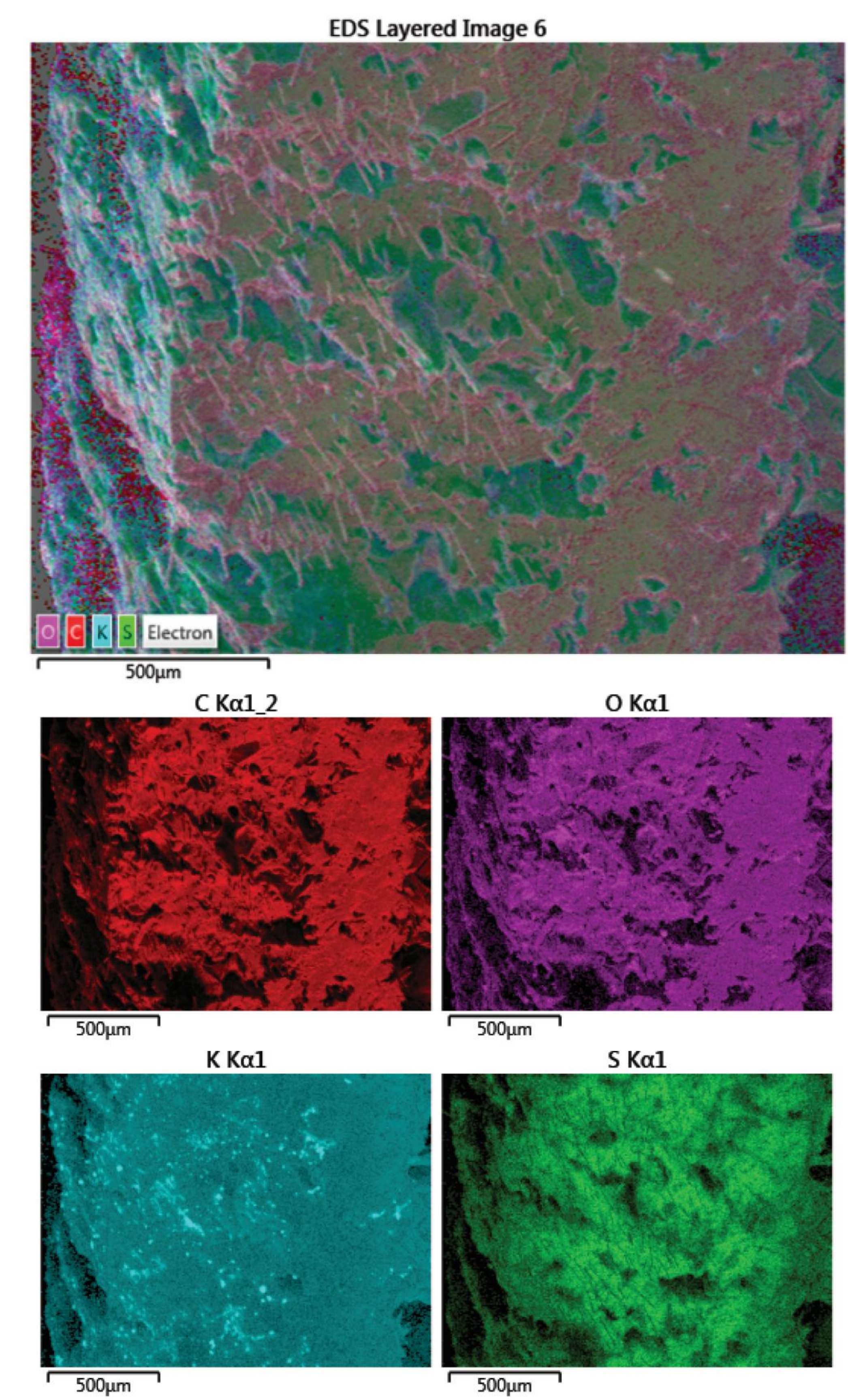

Figure S14. *EDX mapping of the reactor cross-section 3D printed with CF-nylon after 7 cycles of reaction of vinyl derivative synthesis (total reaction time is 11 h at 130 °C)*
